# Digital Health: Tracking Physiomes and Activity Using Wearable Biosensors Reveals Useful Health-Related Information

**DOI:** 10.1371/journal.pbio.2001402

**Published:** 2017-01-12

**Authors:** Xiao Li, Jessilyn Dunn, Denis Salins, Gao Zhou, Wenyu Zhou, Sophia Miryam Schüssler-Fiorenza Rose, Dalia Perelman, Elizabeth Colbert, Ryan Runge, Shannon Rego, Ria Sonecha, Somalee Datta, Tracey McLaughlin, Michael P. Snyder

**Affiliations:** 1 Department of Genetics, Stanford University School of Medicine, Stanford, California, United States of America; 2 Mobilize Center, Stanford University, Palo Alto, California, United States of America; 3 Spinal Cord Injury Service, Veterans Affairs Palo Alto Health Care System, Palo Alto, California, United States of America; 4 Department of Neurosurgery, Stanford University School of Medicine, Stanford, California, United States of America; 5 Division of Endocrinology, Stanford University School of Medicine, Stanford, California, United States of America; Newcastle University, United Kingdom of Great Britain and Northern Ireland

## Abstract

A new wave of portable biosensors allows frequent measurement of health-related physiology. We investigated the use of these devices to monitor human physiological changes during various activities and their role in managing health and diagnosing and analyzing disease. By recording over 250,000 daily measurements for up to 43 individuals, we found personalized circadian differences in physiological parameters, replicating previous physiological findings. Interestingly, we found striking changes in particular environments, such as airline flights (decreased peripheral capillary oxygen saturation [SpO_2_] and increased radiation exposure). These events are associated with physiological macro-phenotypes such as fatigue, providing a strong association between reduced pressure/oxygen and fatigue on high-altitude flights. Importantly, we combined biosensor information with frequent medical measurements and made two important observations: First, wearable devices were useful in identification of early signs of Lyme disease and inflammatory responses; we used this information to develop a personalized, activity-based normalization framework to identify abnormal physiological signals from longitudinal data for facile disease detection. Second, wearables distinguish physiological differences between insulin-sensitive and -resistant individuals. Overall, these results indicate that portable biosensors provide useful information for monitoring personal activities and physiology and are likely to play an important role in managing health and enabling affordable health care access to groups traditionally limited by socioeconomic class or remote geography.

## Author Summary

A new wave of wearable sensors allows frequent and continuous measurements of body functions (physiology), including heart rate, skin temperature, blood oxygen levels, and physical activity. We investigated the ability of wearable sensors to follow physiological changes that occur over the course of a day, during illness and other activities. Data from these sensors revealed personalized differences in daily patterns of activities. Interestingly, we discovered striking changes in particular environments such as airline flights. Blood oxygen levels decreased during high-altitude flights, and this decrease was associated with fatigue. By combining sensor information with frequent medical measurements, we made two important health-related observations. First, wearable sensors were useful in identifying the onset of Lyme disease and inflammation. From this observation, we then developed a computational algorithm for personalized disease detection using such sensors. Second, we found that wearable sensors can reveal physiological differences between insulin-sensitive and insulin-resistant individuals, raising the possibility that these sensors could help detect risk for type 2 diabetes. Overall, these results indicate that the information provided by wearable sensors is physiologically meaningful and actionable. Wearable sensors are likely to play an important role in managing health.

## Introduction

Physiological parameters such as heart rate (HR), blood pressure, and body temperature can provide critical information about the physical health status of a person. Elevation of any of these parameters can be of concern; elevated HR and blood pressure are associated with cardiovascular disease, and elevated body temperature occurs during pathogen infection and inflammation [1–4]. Peripheral capillary oxygen saturation (SpO_2_) is a measure of oxygen saturation of hemoglobin in the blood, and patients with chronic pulmonary disease often have lower resting SpO_2_ and are required to use supplementary oxygen to attain a more optimal SpO_2_ [5]. Skin temperature is associated with alertness levels and quality of sleep [6,7]. Although these different parameters are routinely measured in the physician’s office, they are not generally monitored outside of that context.

The infrequent collection of these measurements as currently practiced is problematic. First, changes in these parameters may not be identified until many months after an initial health condition has occurred. For instance, if a healthy person with reasonable health care access visits his or her physician every 2 y for a routine visit, then a condition may arise many months, or even longer, prior to a clinical symptom onset and thus go undetected for some time. Second, physiological parameters vary among individuals depending on their gender, life stage, and physical training, among other characteristics (e.g., [8,9]). These parameters also vary within the same person during their daily activities and with changes in the ambient environment. Because sparse clinical measurements of an individual are often compared to the average measurements of a population, the large variation within and among individuals results in a difficult medical assessment. Thus, infrequent short measurement periods or lack of adequate health care access makes it difficult to ascertain if a significant health change has occurred in a particular person. This information is particularly valuable for caregivers responsible for the health of others.

Emerging wearable biosensors (hereafter called “wearables”) are a low-cost technology that either continuously or frequently measures physiological parameters and provides a promising approach to routinely monitor personalized physiological measurements and potentially identify alterations in health conditions. Wearables are capable of passive and routine recording and immediate delivery of multiple types of measurements in real time to the wearer or physician with minimal attention or training required. In addition to physiological measurements such as HR and skin temperature, wearable technology has the potential to precisely capture the wearer’s daily physical activities, such as walking, biking, running, and other activities, often in conjunction with a GPS, which provides direct information about the location of the activity.

The popularity of wearable devices has substantially increased in recent years. As of July 2015, there are more than 500 different health care-related wearables present on the market and over 34.3 million devices sold. This is triple the number sold in 2013 [10].

Despite the revolution of wearable technology, studies to investigate their use in health care have been limited. One recent study using biosensors found no obvious benefit to users in health care costs or utilization [11]. In this work, we investigate the use of portable devices to (1) easily and accurately record physiological measurements in individuals in real time (or at high frequency), (2) quantify daily patterns and reveal interesting physiological responses to different circadian cycles and environmental conditions, (3) identify personalized baseline norms and differences among individuals, (4) detect differences in health states among individuals (e.g., people with diabetes versus people without diabetes), and (5) detect inflammatory responses and assist in medical diagnosis at the early phase of disease development, thereby potentially impacting medical care. In addition to a number of novel observations, through these analyses, we have gained considerable insight into the capabilities and value of these different devices in health and scientific research.

## Results

### Overview of the Approach

**Fig 1:**
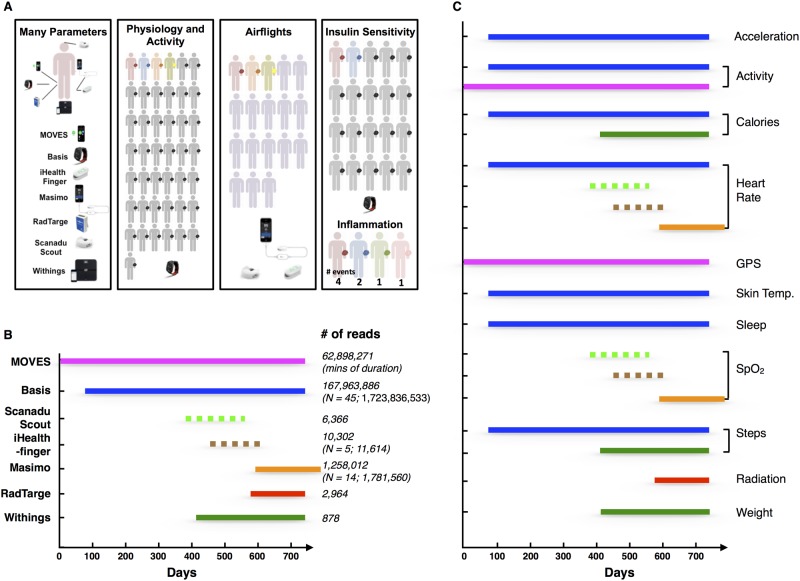
Overview of the project and summary of the devices. (A) Wearable devices used in this study. The different colors for the human figures indicate the specific studies in which each individual participated (i.e., red participated in all five studies, grey in two studies [Physiology/Activity and Insulin Sensitivity], blue in three studies [Physiology/Activity, Insulin Sensitivity, and Inflammation], orange and yellow in two studies [Physiology/Activity and Airflights], and green and pink in one study [Inflammation] and purple in one study [Airflights]). (B) The period during which the devices were used. The number of data points available for Participant #1 and others is indicated to the right. (C) The specific parameters measured by the different devices. The devices used to measure these parameters were represented by the color of the lines (MOVES: magenta; Basis: dark blue; Scanadu Scout: light green; iHealth-finger: brown; Masimo: orange; RadTarge: red; Withings: dark green). Dashed line indicates devices used frequently for discrete measurements; solid lines indicate devices that provide continuous measurement.

Our strategy was to intensely study one individual with many devices in order to determine the ease of collecting different types of data and to identify interesting patterns and then extend our analyses to a cohort of participants using a more limited number of devices (Fig 1A). We began by routinely measuring a 58-y-old male (Participant #1) over the course of 24 mo (Institutional Review Board [IRB] protocols IRB-23602, IRB-34907). From an extensive list of candidate devices, we selected seven that were easy to use, had reasonable accuracy, and had a direct interface for raw data access (see Material and Methods). The list of devices and their measurements is presented in Fig 1. These devices collectively measure (a) three physiological parameters, including HR, SpO_2_, and skin temperature, (b) six activity-related parameters, including sleep, steps, walking, biking, running, calories, and acceleration forces caused by movement, (c) weight, and (d) total gamma and X-ray radiation exposure. Collectively, these devices record more than 250,000 measurements each day (Fig 1). Many of the devices measure the same parameters, enabling cross-device comparison and assessment of measurement accuracy. During this period, Participant #1 also recorded the activities and travel in real time using web-based and smartphone-based software (see Material and Methods for details). Through numerous airline flights (S2A Table), an extensive analysis of the effects of air travel was assessed. Most information was collected through a smartphone, and all data were stored in a common database. Importantly, during the 2-y monitoring period, the participant was extensively monitored (73 visits) with standard medical tests, enabling a detailed comparison of medical data to the wearables information (S3 Table summarizes the medical tests performed in each examination).

In addition to a comprehensive analysis of a single individual, we also analyzed a larger group of participants to examine the consistency of our findings and explore differences and similarities across individuals (Fig 1A; IRB-23602, IRB-34907). Eighteen participants (ages 28 to 72; IRB-34907, whose enrollment criteria were all individuals ages 13 and older were eligible, see Materials and Methods) were analyzed for the effects of airline flight on SpO_2_ levels (IRB-34907; S1B Table). Our analysis of personal baseline and health also included physical monitoring of 43 individuals ages 35 to 70 y using a Basis device for up to 11 mo (average of 152 d; IRB-23602 and IRB-34907; individuals 18 or older were eligible, with a preference for those at risk for type 2 diabetes [T2D]; see Materials and Methods). The latter group is not T2D as defined by fasting plasma glucose <125 mg/dL and is free of chronic inflammatory conditions and major organ diseases. Four individuals, who self-reported as ill and had Basis Peak devices, were analyzed for the capacity of wearables to facilitate illness diagnosis and monitoring. Twenty individuals had quantification of systemic insulin resistance (IR) via the steady-state plasma glucose (SSPG) test as originally described and validated [12,13], and twelve of those individuals were classified as insulin resistant (S1A Table; SSPG greater than 140 mg/dL [14,15]). All together, over 1,788,538,186 measurements from 7,234 d were recorded.

### Summary and Validation of the Devices

Several of the devices, including the Basis device, used most frequently in our study had been validated for clinical-grade accuracy by the manufacturer (See Materials and Methods for details). Nonetheless, we performed extensive testing to assess the accuracy of the different devices against gold standard measurements and/or our instrument (Welch Allyn [WA] 6000 series), which is routinely used at the clinical laboratory services at Stanford University. We found that HR and SpO_2_ data collected using four devices (Scanadu Scout, iHealth-finger, Masimo, and Basis) were very close to that of the WA instrument over a wide range of values using the Bland–Altman method of comparison [16,17] and the Pearson correlation test (see S1 Fig). For example, HR measurements were within five beats per minute (BPM) and 10% of the WA instrument for all devices. SpO_2_ measurements were within 3% for all devices except for the Scanadu, which still yielded similar trends (see Material and Methods). Similarly we found that activity measurements were also close to standards for the conditions measured (e.g., MOVES App: steps: 0.79 +/- 0.16 standard deviation [SD] of the actual value; running: 0.96 +/- 0.05 SD of the actual value; details for all methods are presented in Material and Methods). Thus, we deemed the wearable biosensor measurements to be suitable for these studies.

### Circadian and Diurnal Patterns in Physiological Parameters

**Fig 2:**
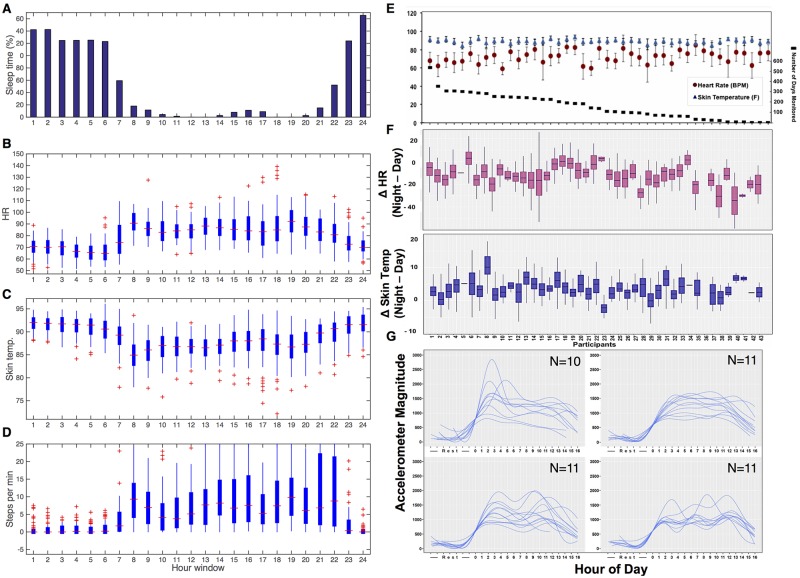
Circadian and diurnal patterns in physiological parameters. Participant #1 hourly summaries in (A) sleep, (B) HRs, (C) skin temperature, and (D) steps as measured using the Basis Peak device over 71 nontravel d. (E) Summaries of 43-person cohort for daily HR and skin temperature from all data and (F) differences in resting (fewer than five steps) nighttime and daytime HRs (Note: one person did not have nighttime measurements and is not included) and skin temperature. (G) Daily activity plots for 43 individuals. Based on number of peaks in the curves, four general patterns of activity behavior are evident. The plots in Fig 2G were aligned according to the first increase in activity.

In order to understand deviation from normal patterns, we first analyzed the collected data for systematic normal patterns, such as circadian rhythms, beginning with Participant #1. To reduce effects due to travel, our analyses focused on days lacking distance travel (defined as trips taken using airlines, assessed using GPS data from MOVES, and validated by comprehensive personal logs/calendars; see Material and Methods). Fig 2A–2D shows the circadian patterns of HR, skin temperature, and activity for 71 nontraveling d of Participant #1. As expected, we detected clear cyclical fluctuations over 24-h periods. For example, HR (measured using the Basis Peak) is generally lower at night (mean of 69.2 +/- 7.7 SD BPM from 10 p.m. to 6 a.m.) and higher during the day (mean of 84.5 +/- 11.3 SD BPM from 6 a.m. to 10 p.m.), with daily fluctuations (peak/trough or max/min) of 46.4 +/- 11.6 SD BPM (Fig 2B, S2A Fig), consistent with the sleep–wake cycle indicated by the Basis device (Fig 2A). Skin temperature measurements also generally followed a similar day-and-night pattern. Unlike that reported for core temperature [18], we found that skin temperature increases during sleep (a mean of 91.3 +/- 2.0°F for 10 p.m. to 6 a.m.; a mean of 86.6 +/- 3.2°F for 6 a.m. to 10 p.m., with daily fluctuations of 11.5 +/- 2.9 SD°F on average; Fig 2C, S2B Fig).

Comparison of physiological data with physical activity information revealed obvious activity-related physiological responses during specific time windows. Participant #1 often has an elevated HR during the 7 a.m.-to-8 a.m. and 6 p.m.-to-7 p.m. time windows, which included the typical time for bike commuting on weekdays (confirmed with daily calendar and consistent with MOVES information). On weekends, elevated HR was often evident in the 4 p.m.-to-6 p.m. window (S2 Fig), which is consistent with running activity measured using both the Basis device and MOVES. Overall, the correspondence of patterns detected with known activities indicates that the wearable devices can readily capture physiological information.

### Physiological Parameters Change Dynamically with Human Activity

We also directly compared the physiological response in relation to different daily activities using data from the Basis device and MOVES apps (see Material and Methods). As shown in S3 Fig, our results replicate well-known patterns of physiological responses to events [19–22], including significantly faster HRs during exercise and significantly slower HRs during sleep compared to activity-free times, the mean of which is 78.4 ± 14.7 (SD) BPM; a mean of 67.6 ± 8.3 (SD) BPM, 101.1 ± 15.4 (SD) BPM, 114.1 ± 14.1 (SD) BPM, and 145.2 ± 18.1 (SD) BPM were observed during sleep, walking, cycling, and running, respectively (two-sided Wilcoxon rank sum *p* < 10^−32^). As expected, the measurements of HR, steps, calories, and skin temperature are very consistent for most of the activities, except the step measurement during cycling, which is not accurately detected using the Basis device (S3 Fig). Importantly, as described below, examination of recorded notes revealed a significant decrease in SpO_2_ levels measured by both the forehead and finger devices when Participant #1 reported fatigue (two-sided Wilcoxon rank-sum test *p* < 0.05; see below), and this finding was validated using systematic fatigue testing as described in the section on Airline Flights. Overall, these results indicate that our devices capture data as expected and also serve as a useful baseline to detect outlying measurements, as described below.

### Personalized Physiological and Activity Profiles for 43 Individuals

**Fig 3:**
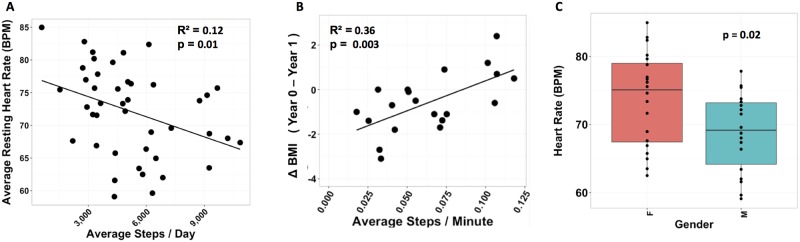
Physiological and activity profiles for 43 individuals. (A) The relationship between the average number of steps per day and resting HR (*n* = 43) and (B) average steps per minute and change in body mass index (BMI; *n* = 20) over the course of approximately 1 y was analyzed. Average resting HRs (C) were calculated by gender (see Material and Methods; *n* = 38).

We further examined physiological parameters and activity patterns for 43 participants, including Participant #1, who wore a Basis device for between 1 and 24 mo. For the overall cohort, the resting HR was 72.10 ± 6.75 (SD) BPM and the resting skin temperature was 89.19 ± 1.88 (SD)°F. We found a significant difference in resting HR between men and women (Fig 3C): the resting HR of women was 73.70 BPM versus 68.80 BPM for men (*p* = 0.02248, Welch two-sample *t* test with 95% CI). These values are very similar to those reported by the NHANES study (74 BPM for women; 71 BPM for men) [23]. Women in our cohort also have a slightly higher average skin temperature (89.6°F) than men (88.7°F), but the value did not reach significance (*p* = 0.1724, Welch two-sample *t* test with 95% CI). In general, and as expected, we find that HR increases (S4A and S4B Fig) and skin temperature consistently decreases (S4C Fig) with increasing activity [21,22]. Furthermore, we see that the relationship between HR and skin temperature varies considerably among individuals (S4 Fig).

We analyzed the changes in HR and skin temperature between day and night for each of the 43 additional participants, although one individual (Participant #36) did not wear the Basis device while sleeping and was excluded from this analysis (Fig 2F). Average daytime HR (79.48 ± 6.96 BPM) was significantly higher than nighttime HR (66.99 ± 8.04 BPM; *p* = 4.836e-13, paired *t* test with 95% CI). The average difference between daytime and nighttime HR was 12.50 ± 7.80 BPM. Average daytime skin temperature (88.02 ± 2.02°F) was significantly lower than nighttime skin temperature (91.49 ± 1.77°F; *p* = 4.87e-13, paired t-test with 95% CI). The average difference between daytime and nighttime skin temperatures was 3.47 ± 2.17°F. This is consistent with findings that skin temperature increases by 7.2°F (winter) and 5.4°F (summer) during sleep [24].

As shown in Fig 2E, the differences in resting HR and skin temperature values differ widely among individuals. For resting HR, the values vary from 59.09 ± 6.59 to 84.97 ± 11.29 BPM. The range of values for skin temperature is smaller than that of HR, with values of 84.44 ± 3.85 to 93.65 ± 2.05°F, indicating tighter regulation of this physiological parameter. Although the Basis device has two sensors for detecting skin and ambient temperature, it is still possible that differences in skin temperature across individuals could be due to technical considerations in how the device is worn and/or exposed (S1C Table); however, the considerable differences in diurnal and nocturnal resting HR are likely to be due to personal differences between individuals because any measurement bias caused by how the device was worn is removed through differencing of daytime and nighttime values. Overall, we did not detect an obvious correlation between HR and skin temperature; this might be due to complications from the diverse activities of the individuals.

We also examined the activity patterns of the 43 individuals using the Basis device data. The individuals fell into four major groups, including individuals with highest activity in the early morning (Morning Active; Fig 2G, upper left panel), sustained activity during the day (All Day Active; Fig 2G, upper right panel), or peaks in activity either two times (Commuter Active; Fig 2G, lower left panel) or three times (Mealtimes Active; Fig 2G, lower right panel) daily. The peaks for the latter two categories fall between mealtimes (i.e., mid-morning, mid-afternoon, and, for one group, in the evening). We used this finding to train functional clustering machine learning algorithms to classify individuals by activity group (S2 Fig).

Because increased activity is associated with overall fitness levels, we examined the relationship between activity, resting HR, and weight loss. We observed that a higher average number of steps per day is associated with lower resting HR (adjusted R^2^ = 0.12, *p* = 0.01462; Fig 3A), and, when following changes over the course of 1 y, the increased average steps per minute is associated with a decrease in body mass index (BMI; adjusted R^2^ = 0.36, *p* = 0.003058; Fig 3B). In general, individuals with overall higher activity levels have less of a change in HR between high and low activity periods (S4B Fig), indicating increased fitness levels. Overall, these result indicate that there are highly varied baseline physiological differences as well as activity differences among individuals that relate directly to clinically relevant parameters, suggesting that individuals have personal physiome and activity patterns that can be tracked using wearable sensors.

### Significantly altered Physiology during Airline Flights

From our detailed analysis of Participant #1, a striking and interesting change in physiological measurements was observed during airline flights, and, consequently, we pursued an in-depth analysis of physiological parameters during air travel. Cabin pressure in an aircraft is normally maintained at a reduced level with a minimum value comparable to that of 8,000 feet altitude [25], although several modern aircraft have been advertised to maintain higher cabin pressure. For Participant #1, we measured SpO_2_ and HR for 96 flights (summarized in S2A Table). The length of the flights varied from 23 min to 829 min, with 40 short flights (<2 h), 39 median-length flights (2–7 h), and 17 long flights (>7 h). Thirteen different aircraft models were included. The SpO_2_ level of Participant #1 was monitored by a forehead device (Scanadu) and/or finger monitoring devices (iHealth-finger, Masimo) in a continuous (Masimo) or discontinuous manner (Scanadu, iHealth-finger). The FlightAware website (https://flightaware.com/) was used to track specific details about the plane routing, altitude, and speed for each flight in real time.

**Fig 4:**
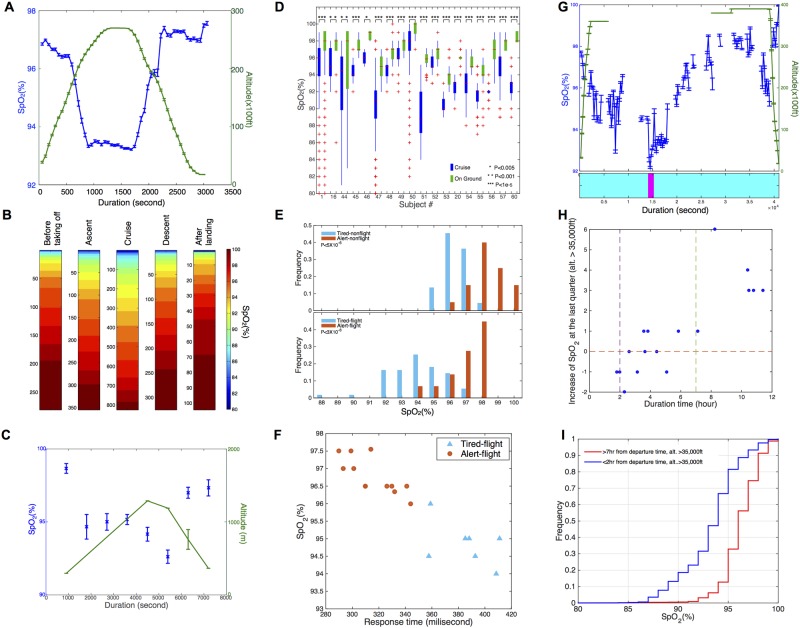
SpO_2_ measurements during flight. (A) Example of a flight with continuous SpO_2_ measurements (blue) taken using a Masimo finger device. Altitude recorded using FlightAware (green). (B) Heat map showing distribution of SpO_2_ measurements recorded using a forehead Scanadu device at different flight stages: before takeoff, ascending, cruising, descending, and on ground post flight. (C) SpO_2_ levels recorded using iHealth-finger device during 2-h automobile ride over a mountain. Average measurements and standard error measured over a 15-min window (Blue). Altitude recorded from sign markers or town elevations and/or using DraftLogic website. (D) Distribution of SpO_2_ measurements taken from 18 individuals at cruising altitude (blue) versus on ground (green). (E) Distribution of SpO_2_ measurements after the participant reported feeling alert (red) or tired (cyan). (Upper panel) Measurements from nonflying days. (Lower panel) Measurements from flying days. The significance of the difference between the two distributions was assessed by two-sample Kolmogorov–Smirnov test. (F) Scatterplot of response time and SpO_2_ level recorded during one flight. The data recorded during another flight are shown in S5D Fig. The response time was derived from the psychomotor vigilance test to objectively quantify the fatigue of the participant. Self-reported tired and alert states are labeled by cyan triangles and red dots, respectively. (G) (Upper panel) Example of a flight with continuous SpO_2_ measurements (blue) taken using a Masimo finger device. Altitude recorded using FlightAware (green). Note the increase in SpO_2_ level towards the end of the flight. (Lower panel) Sleepiness recorded by Basis device. Magenta and cyan colors represent sleep and awake status, respectively. (H) A scatterplot of duration of time and the increase of SpO_2_ in the last quarter. All data points were collected at altitudes higher than 35,000 feet. (I) Empirical cumulative distribution function plot of SpO_2_ levels >7 h after takeoff (red) versus <2 h after takeoff (blue). All the data points were recorded at altitudes higher than 35,000 feet (*p* < 1e-307; two-sample Kolmogorov–Smirnov test).

We observed a striking decrease in SpO_2_ levels during airplane flights (a typical flight is shown in Fig 4A and S5A Fig), and this decrease is strongly negatively correlated with altitude. To summarize all flights, we binned each flight into five stages: before takeoff, ascent, cruise, descent, and after-landing stages (see Material and Methods). The overall distribution is shown in Fig 4B and S5B Fig for Scanadu and iHealth-finger measurements, respectively. Notably, Scanadu-measured SpO_2_ levels were at 97%–100%, 91%–96%, and 90% or less for 31.2%, 54.0%, and 14.8% of measurements, respectively, in the cruising stage as compared to 64.1%, 31.7%, and 4.3% in the stage prior to takeoff and 73.1%, 24.0%, and 2.9% in the after-landing stage (Fig 4B); iHealth-finger-measured SpO_2_ levels were at 97%–100%, 91%–96%, and 90% or less for 29.5%, 65.4%, and 5.1% measurements, respectively, in the cruising stage as compared to 88.0%, 10.8%, and 1.2% in the before takeoff stage and 80.6%, 13.9%, and 5.6% in the after-landing stage (S5B Fig). For the first 20 Masimo-measured flights, 19 had a significant inverse correlation of SpO_2_ levels with altitude (*p* < 4e-47 for each flight; the remaining flight had technical issues, see Material and Methods for details; see also S6A and S6B Fig for an aggregate SpO_2_ versus altitude of all flights, *p* < 1e-307). Thus, regardless of the device and flight, SpO_2_ levels correlate inversely with altitude. Seating locations were not found to have an effect on SpO_2_ levels. Overall, we observed a drop to a SpO_2_ of 96% or lower in all flights, and in many cases the drop was quite low (less than 94%) for a significant portion of the flight.

We hypothesized that the SpO_2_ reduction is most likely due to the reduced air pressure, leading to reduced available oxygen at cabin altitudes. To evaluate whether the reduced pressure is the primary cause versus other flight-related factors, we also measured SpO_2_ levels on a ~2 h automobile trip that climbed 993 meters and decreased 924 meters (altitude was determined by personal logs and the DraftLogic website https://www.daftlogic.com/sandbox-google-maps-find-altitude.htm; Fig 4C). SpO_2_ levels tightly correlated with altitude, indicating a direct relationship between SpO_2_ level and air pressure/oxygen [26].

Interestingly, we observed that on long flights the SpO_2_ levels are higher toward the end of the flight than those at the beginning (a typical flight in shown in Fig 4G; data for a number of long flights are shown in S6 Fig). Among the 17 Masimo continuous recorded flights that have records for the last quarter of the flight, we observed that on long flights greater than 7 h, SpO_2_ levels in the last quarter are significantly higher than at least one of the other three quarters measured at the same altitude level (Wilcoxon ranksum test *p* < 1x10^-29^). and this observation was not detected on the short flights (Fig 4H). Furthermore, if we binned the Masimo-recorded, high-altitude (>35,000 feet) SpO_2_ levels into two categories, (1) measured after 7 h from departure time and (2) measured earlier than 2 h from departure time, we observed significantly higher SpO_2_ levels in the former category compared to the latter one. This increase is likely due to either adaptation or a physiological change after rest/inactivity. For the long westward flights, Basis-measured activity was relatively constant (i.e., primarily sitting >95% of the flight) and with little or no sleep (Basis-quantified and self-reported); an example is shown in Fig 4G, and the SpO_2_ increase at the end of flight was always observed (see Fig 4G–4I and S6 Fig; Scanadu measurements are in S5F Fig), indicating that this increase is most likely due to adaptation. This observation demonstrates that humans can adapt to low oxygen after a number of hours on an aircraft.

To determine how SpO_2_ measurements relate to macrophenotypes, we also examined physiological measurements during periods when the participant logged their alertness status as either “tired” or “alert” using a blind scoring system (its calibration has been quantified as described in Material and Methods). As shown in Fig 4E (bottom panel), the SpO_2_ level reported when “tired” on flights was significantly lower compared to that measured when “alert” (two sample Kolmogorov–Smirnov test *p* < 3x10^-8^), similar to that reported on nonflying days (Fig 4E upper panel, two-sample Kolmogorov–Smirnov test *p* < 5x10^-6^). This observation was evident by both finger (Fig 4E) and forehead devices (S5C Fig). To be more quantitative in reporting fatigue, the participant also performed a psychomotor vigilance test and quantified fatigue by measuring the speed with which participants respond to a visual stimulus (see Methods and Material for details). As shown in Fig 4F and S5D Fig, a longer response time was required when the participant logged their state as “tired” rather than “alert,” and the SpO_2_ level is strongly negatively correlated with the response time (Pearson correlation test R = -0.88 to -0.91, *p* < 6x10^-5^). This result not only validates the self-reported system of fatigue but also provides a quantitative summary of the relationship between fatigue and SpO_2_ level. Although reductions in SpO_2_ levels during flights have been reported previously [27–32], to our knowledge, this the first report of (a) adaptation on long flights and (b) fatigue levels on actual commercial aircraft with objective assessment of fatigue (see Discussion).

To further examine whether oxygen levels decreased in other individuals during airplane flights, we measured SpO_2_ levels in 17 other individuals from diverse ethnic backgrounds (European, Jewish, African American, Indian Asian, and East Asian) using the iHealth-finger device or Masimo device (details in Material and Methods, S1B and S2B Tables). In every case, decreased SpO_2_ levels were observed during cruising (difference between median SpO_2_ varies from 2% to 9%, two-sided Wilcoxon rank sum test *p* < 0.001). We also found that baseline SpO_2_ and decrease in SpO_2_ during cruising varies with different individuals (Fig 4D). A plot of altitude versus SpO_2_ reveals that, in general, SpO_2_ decreases are lower at lower-altitudes flights, and this is particularly evident for individuals with more than four flights (S6 Fig). Overall, these results indicate that reduced SpO_2_ levels during air travel are a general phenomenon and occur in all types of aircraft. This result is consistent with published observations [27–32] and further indicates that the decreased SpO_2_ during air travel is evident across different ethnic groups.

### Diagnosis of Diseases Associated with Inflammation

To investigate the capacity of wearables to facilitate disease diagnosis and monitoring, we examined the association between unusual physiological signals and disease status or disease markers. This was uniquely possible for our study because we had frequently sampled and performed a number of biomedical assays during the entire monitoring period (see S3 Table for the list of tests).

**Fig 5:**
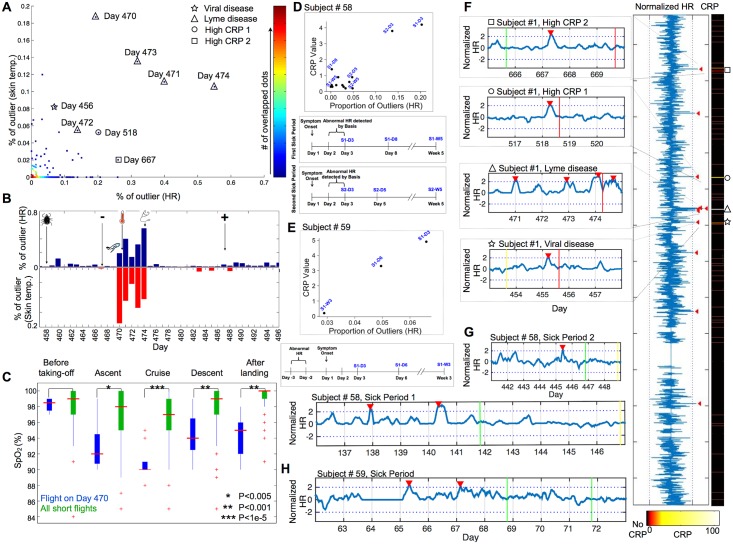
Elevated physiological measurements during infections. (A) Plot of fraction of outlying skin temperatures and HRs for all 679 d of Participant #1. Note all outlying time points correspond to periods when elevated high-sensitivity C-reactive protein (hs-CRP) measurements and/or illness were noted. The period harboring Lyme disease is expanded in panel B. (C) Decreased SpO_2_ measurements during the flight and subsequent period when aberrant physiological measurements were first noted; boxplot shows SpO_2_ distribution on Day 470 flight (blue) relative to similar length flights (green). The significance of this difference was assessed by two-sided Wilcoxon rank sum test. (D, E) CRP measurements are plotted against the proportion of daily HR measurements that were more than two SDs above the mean for Participant #58 (Pearson correlation coefficient = 0.90, *p* = 1.066e-05) (D) and Participant #59 (Pearson correlation coefficient = 0.966, *p* = 0.1653) (E). The timelines for the illness progression, CRP measurements, and Basis monitoring period captured in the figure are indicated for Participant #58 (two different illnesses separated by a period of ~11 mo) (Lower panel of D) and Participant #59 (Lower panel of E). (F) 679-d monitoring period of Participant #1. Left: normalized HR in minute resolution. Zoomed in at each illness period. Right: elevated CRP periods; G-H. Normalized HR at sick periods in minute resolution for Participant #58 (G) and Participant #59 (H). Red peak: Abnormal periods indicated by the peak caller. Red vertical line: CRP larger than 10; Green vertical line: CRP larger than three but smaller than ten. Yellow line: CRP smaller than three.

We began our analysis by focusing on Participant #1, who was measured continuously for HR and skin temperature and frequently for SpO_2_ levels for a period of 679 d (measurements were recorded for 603 d; see Material and Methods) and had a very large number of days (73) with extensive clinical testing during this period. As indicated above, physiological parameters change dynamically with daily activities (e.g., significantly faster HRs during exercise [S3 and S4A Figs] and significantly slower HRs during sleep [S3 Fig, Fig 2B and 2F]). Therefore, we compared each parameter according to the corresponding activity information (see Material and Methods for details) and chose those periods lacking physical activity to calculate the percentage-of-outliers (i.e., percentage of reads per day classified as outliers from the overall personal mean) to search for periods with significantly different measurements. During the 603 d of monitoring, we identified 8 d with abnormal HR and skin temperature pattern (Fig 5A; S7A Fig). Interestingly, most of these days fell into four periods that are of very high interest from a health perspective.

(1) The most significant period was a 5-d period (Days 470–474), during which we detected abnormally elevated HR (~14% to ~55% reads per day were defined as significant outliers compared to the corresponding baseline norm) and skin temperature (~5% to ~19% of the reads per day were defined as significant outliers compared to the corresponding baseline norm) during each of these days (Fig 5A and 5B). During this period, Participant #1 was suffering from Lyme disease (as diagnosed on Day 487 by a positive antibody test, S7D Fig). Lyme disease is a *Borrelia* bacterial infection primarily transmitted to humans through tick bites; 12 d prior to this period (on Day 458), the participant had been exposed to an area in rural Massachusetts where high levels of Lyme-infected ticks are present. (Note that a “bull’s eye” rash was not observed during the initial period of infection). Importantly, the participant first noticed a possible health concern at the onset of the elevated HR/skin temperature period (on Day 470) and by abnormally low SpO_2_ readings both during an airline flight and afterwards (Fig 5C). This elevated condition was followed over the next 4 d by a persistent elevated temperature reading with an oral thermometer (98.9–102°F; see Material and Methods for details). Importantly, the normalized HR and skin temperature from the wearable device were significantly elevated multiple times during this period, and the HR exhibited a strong correlation with measurements taken with the oral thermometer over this period (R = 0.81, *p* <0.05, S7B Fig). The participant visited a physician at Day 474 and received treatment with doxycycline; the symptoms and abnormal vital signs disappeared the following day (Fig 5B).

(2) Another interesting period of outlying HR and skin temperature was Day 518 (Fig 5A, S7A Fig). About 20% of the HR reads and about 5% of the skin temperature reads on that day were defined as outliers compared to the corresponding baseline norm. Importantly, an elevated high-sensitivity C-reactive protein (hs-CRP) level was detected that day by a blood test (hs-CRP: 24.8 mg/L; baseline is normally <0.2 mg/L), although clinical symptoms (i.e., fever) were not reported. The elevated CRP, HR, and skin temperature were identified during data analysis.

(3) Another interesting period is from Day 455 to Day 456 (Fig 5A, S7A Fig). About 16% of the HR reads on Day 455 and about 8% of the skin temperature reads on Day 456 were classified as outlier compared to the corresponding baseline norm. Importantly, Participant #1 was diagnosed with a Human rhinovirus infection during this period (GeneMark test) and had elevated hs-CRP levels and inflammatory cell counts (hs-CRP: 16.2 mg/L, white blood cell: 10.8 K/ul, Neutrophil: 8.4 K/ul, 77.9%).

(4) Another interesting period is from Day 665 to Day 669 (Fig 5A). About 26% of the HR reads on Day 667 and about 2% of skin temperature reads were defined as outliers compared to the corresponding baseline norm. Importantly, an elevated hs-CRP level was detected on Days 665 and 669 by a blood test (hs-CRP: 4.3 mg/L and 15.4 mg/L on Days 665 and 669, respectively). The participant reported congestion during this period.

A plot of outlying HR and skin temperature as a function of time during each of these periods further demarcates their deviation from baseline and illustrates that the Lyme disease period has the highest outlying measurements (S8A Fig).

In summary, each of the circumstances with both elevated outlying HR and skin temperature was associated with elevated hs-CRP, indicative of a high inflammatory response (Fig 5A, S7 Fig), and for three of the four periods, clinical symptoms were reported. The participant did not report any other illness during this period of monitoring. These data indicate that there is a strong correlation between inflammatory response and elevated HR and skin temperature, which can be detected by wearables (Pearson correlation coefficient for CRP and the fraction-of-outlying-heart-rate (R = 0.96, *p* < 10e-28); coefficient for CRP and fraction-of-outlying-skin-temperatures (R = 0.94, *p* < 10e-24). For the case of Lyme disease, the abnormal physiological measurements of SpO_2_ and HR were important in alerting the participant to the disease.

To determine whether disease-associated events might be detected in other individuals using wearables, we identified three other individuals in our cohort who self-reported as ill and had Basis devices (but not SpO_2_ measurement devices; Fig 5D and 5E, S7E–S7G Fig). One individual had been ill twice. In each of these four instances, high CRP levels and elevated HRs were evident relative to their personal backgrounds (between 2.02 and 4.66 SDs above background). Although one of the individuals also had elevated skin temperature during this period (S7E Fig), interestingly, for two of the individuals (three illnesses), we did not detect elevated skin temperature. This might relate to differences in how the device was worn, as our survey results demonstrate that the device was worn loosely for at least one of these two individuals. As with Participant #1, for these three individuals, we also searched for other periods of elevated resting HR. For Participant #37, the illness period was the strongest outlier by a very large amount (4.66 SDs above background; rank #1 out of 25 d of monitoring for fraction of HR outlying measurements, S7F and S7G Fig). For Participant #58, the two illness days were in the top 5% of elevated HR outliers (3.40 and 2.02 SDs above background; ranks #10 and #19 out of 568 d of monitoring, Fig 5D), but we do not have the corresponding CRP levels to the other dates with outlying HR to know if those dates represent periods of illness/inflammation. For Participant #59, elevated HR occurred between 48–72 h prior to reported symptoms (3.55 SD above background; rank #1 out of 138 days of monitoring, Fig 5E) and elevated skin temperature on the day of and 48 h prior to reported symptoms (2.15 and 2.45 SDs above background, ranks #4 and #2 of 138 d of monitoring, respectively; S7E Fig).

In summary, we observed elevated HR during each ill period for all four individuals (eight total events), which suggests that monitoring of HR (and sometimes skin temperature) using a wearable device can detect inflammatory periods.

### High-Resolution Mapping of Inflammatory Disease

To examine the resolution at which illness might be confidently identified, we developed a computational approach called “Change-of-Heart” or COH to identify periods with abnormal HR patterns. HR was chosen because, as described above, it reliably detected all periods with elevated CRP levels in each of the individuals. We were unable to reliably map elevated skin temperature at high resolution during these periods across all individuals, and thus this parameter was not pursued. Specifically, we focused on deviations in resting HRs relative to an inactive period and applied a peak-finding–based algorithm to the smoothed continuous HR signal to search for peaks different from a global and local distribution (see Material and Methods). This peak-finding method is optimal for identifying times of transition from healthy to ill states, and thus preferentially detects early periods of infection, which is most desirable.

As shown in Fig 5F, during the 679 d when Participant #1 was monitored, we identified 11 periods with elevated HR. These periods successfully tagged all of the four sick periods indicated above, sometimes with multiple peaks, and also revealed four other periods during which no illness was reported. Application of this approach to the other three individuals also revealed peaks during each of their ill periods. For all four individuals, we are able to identify all of the sick periods using this method with area under the receiver operating characteristic curves larger than 0.9 for each individual (S8B Fig). Importantly, each illness period is identified (100% sensitivity), and for most of the sick periods, significant signals were evident at the very beginning of the illness period. Overall, these results indicate that elevated HRs are present during illness and can be detected using wearable devices.

### Physiological Differences in IR and Insulin Sensitivity are Detectable using Wearables

The availability of clinical measurements on our participants enabled us to investigate associations between information collected from wearables with clinically important data. We focused on diabetes-related measurements because many of our participants were at risk for T2D. Diabetes is a significant rising global health problem, and IR is highly correlated with progression to T2D [33]. Twenty individuals in our cohort underwent measurement of their SSPG, a direct measurement of resistance to insulin-mediated glucose uptake (See Material and Methods) [12,13].

**Fig 6:**
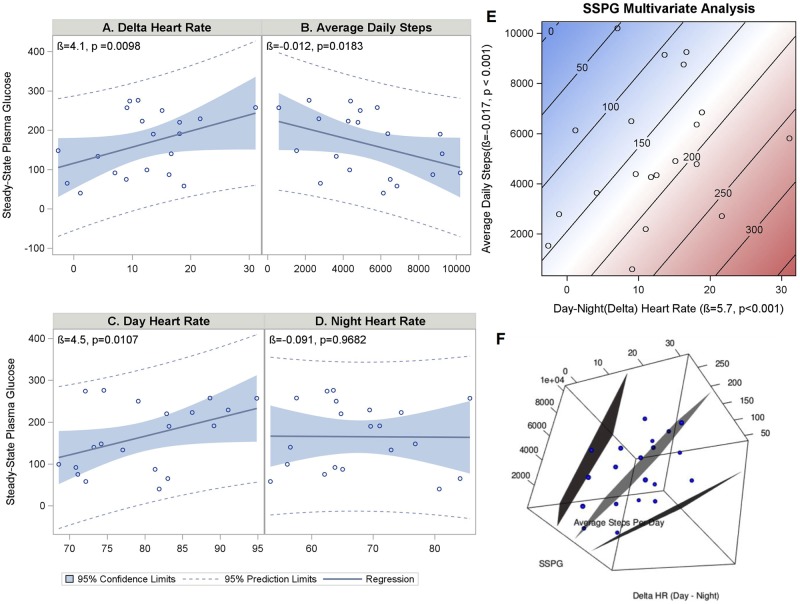
HR differences in IR and sensitivity. Fit plots (A–D) of the linear regression models showing the associations between daytime (C), nighttime (D), delta (daytime minus nighttime) HR (A), and average daily steps (B) with SSPG levels. Higher SSPG levels indicate increased IR. An increase in delta HR and daytime HR is associated with a higher SSPG level, whereas an increase in average number of steps taken a day is associated with a lower SSPG level. There was no association with nighttime HR. Contour fit plot (E) and 3-D plot (F) of the multivariate regression demonstrating that delta HR and average daily steps each have an independent inverse association with SSPG. Parameter estimates were obtained using restricted maximum likelihood estimation with a robust variance estimator to account for unequal variances.

We performed a stepwise modeling approach to examine the relationships between SSPG values and HR, activity, and BMI, beginning with a simple univariate model and then building to bi- and trivariate models. We first examined the associations between daytime, nighttime, and delta (daytime minus nighttime) HR and SSPG (Fig 6A, 6C and 6D) because of evidence that diabetes is associated with changes in diurnal variation of HR [34]. Both daytime HR (Fig 6C) and delta HR (Fig 6A) were positively correlated with SSPG (Daytime HR: β = 4.5, 95% CI 1.2–7.8), *p* = 0.0107; Delta HR: β = 4.1 (95% CI 1.1–7.1), *p* = 0.0098), but nighttime HR (Fig 6D) was not.

Because our previous results showed a relationship between overall activity and resting HR (Fig 3), we wanted to evaluate whether the relationship we discovered between daytime or delta HR and SSPG was due to differences in study participant activity. We first assessed whether there was a relationship between daily activity and SSPG (Fig 6B) and found that average daily steps had an inverse relationship (β = -0.012, 95% CI -0.022–-0.002, *p* = 0.0183) with SSPG. We also evaluated the relationship between average daily steps and HR and found that daily steps was not significantly associated with daytime HR (β = -0.0008, 95% CI -0.0021–0.0005, *p* = 0.1943) but did have a significant inverse relationship with nighttime HR (β = -0.0017, 95% CI -0.0030–-0.0004, *p* = 0.0115). Thus, the association of higher daytime HR with higher SSPG levels is unlikely to be due to differences in participant daily activity. Including overall activity in a multivariate regression model with delta HR to predict SSPG resulted in an improved adjusted R^2^ to 0.41 from 0.17 in the univariate model with delta HR as the only predictor. These results suggest that information from different wearable sensor data types in combination can improve the ability to detect important physiological changes as compared to information from a single sensor.

To assess whether BMI plays a role in the relationship between delta HR and SSPG, we further expanded our multivariate regression to include BMI. BMI is known to have a positive correlation with HR [35], and IR and is negatively correlated with overall activity levels (Kruger et al., 2016). In this model, delta HR remained a strong predictor of SSPG levels (β = 5.05, 95% CI 2.73–7.37, *p* = 0.0003) independent of daily activity (β = -0.010, 95% CI -0.021–0.000, *p* = 0.0509) and BMI (β = 7.58, 95% CI 1.83–13.33, *p* = 0.0130, adjusted R^2^ = 0.52). Thus, combining information from multiple wearable sensors and electronic medical records to capture the relevant underlying physiological parameters enables enhanced prediction of SSPG. Overall, these results indicate that individuals with different degrees of IR and insulin sensitivity have important physiological differences and that these differences can be measured using wearable devices.

### Exposure to Radiation

**Fig 7:**
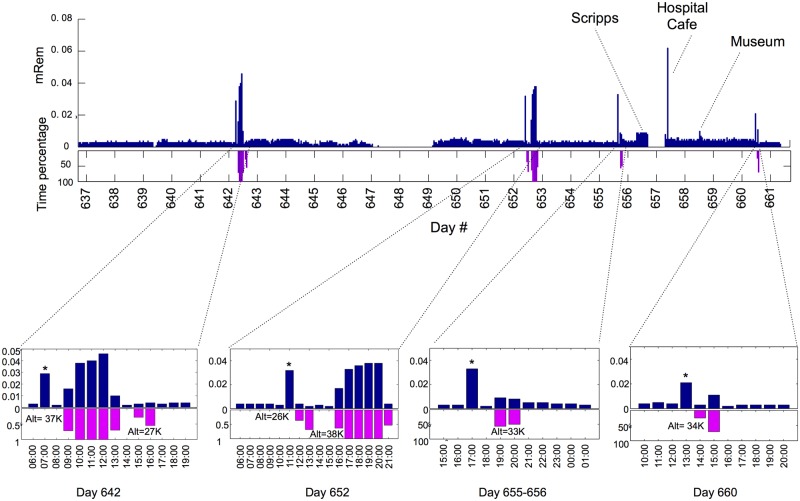
Exposure to radiation in daily life. Bar plot (upper panel: bars in blue) showing the amount of radiation that Participant #1 exposed to over a 25-d time window. Bar plot (lower panel: bars in magenta) showing the time that Participant #1 spent in airplane flights over the same time period. The maximum cruising altitude of each flight was labeled in the zoomed view of the bar plots. Asterisk represents the amount of radiation monitored during the airport carry-on luggage check (range 0.027 to 0.031 mRem). Other events that resulted in relatively high radiation are also labeled in the figure.

Lastly, to examine the diversity of measurements that can be quantified, we also explored whether individuals encounter periods of radiation exposure using a personal radiation tracker (RadTarge II D700) to monitor the local environmental radiation level over a 6-mo period. Fig 7 displays the distribution of radiation exposure over a 25-d period. As suggested by these data, Participant #1 typically lives in an environment with a low background radiation level around 0.003+/-0.0006 millirem (mRem) per h; however, several exposures of elevated radiation levels occurred. The majority (>90%) of the events over 0.030 mRem per h occurred during airplane flights, consistent with the expectation of increased exposure to cosmic radiation at high altitudes [36–38]. As shown in the enlarged panel, the radiation level per h generally corresponds closely with the interval and altitude of the airplane flight (typically rising to 0.038+/-0.004 mRem/h for a 35,000–39,000 feet altitude flight), an increase of 12.7-fold over home background levels). Short flights with lower cruising altitude (a flight on Day 642 with a maximum cruising altitude of 27,000 feet and the one on Day 652 with a maximum cruising altitude of 26,000 feet) yield only modest increases in radiation exposure over the background. The increased exposure at high altitudes is consistent with the well-known fact that a radiation-protective layer of atmosphere surrounds the Earth and is diminished at higher altitudes [39].

Several other interesting periods of increased radiation levels were evident. A very modest increase was evident for an entire 8-h period at the Scripps Research Institute (3-fold) and in an underground museum in Los Angeles (also 3-fold). A very substantial increase (400-fold at peak levels) in higher radiation was detected when the participant entered a hospital café. This increased exposure lasted the entire 10–15-min period in the café but was not evident upon return to the same location two times later in the same day. A likely explanation for this observation is that the radiation source was present on someone in the café, most likely an individual undergoing internal radiation therapy. We did not detect differences in HR or skin temperature during this period. Overall, these data show that very modest increases in radiation are present in several locations in the country with more substantial increases during airline flights or during chance encounters with individuals or locations with high radiation. Most importantly, it demonstrates that simple personal wearable devices can identify these levels and provide immediate feedback.

## Discussion

The data presented above indicate that many types of continuous physiological and activity information can be collected on a single individual on a long-term basis and can be used to measure, analyze, and guide health-related decisions. We showed that wearables can capture expected observations such as circadian fluctuation in HR and skin temperature and their changes during activity. In addition to serving as a valuable resource, we also found several interesting and important new results.

First, we found a decrease in SpO_2_ levels on airplane flights, including the frequent intervals (14.8%) with very low SpO_2_, with an adaptation toward more normal levels on long (>7 h) flights. The former has been reported previously [27–32], but, to our knowledge, the SpO_2_ decrease on modern aircraft (including Boeing 787) and the interesting discovery of SpO_2_ adaptation after approximately 7 h on long flights have not been reported. The SpO_2_ decrease is unlikely to be due to inactivity because similar periods of inactivity not on flights did not associate with SpO_2_ decrease. We suggest that the adaptation is due to altered physiology; it is unclear if frequent flying contributes to the adaptation response.

We also found a significant association of SpO_2_ decrease with fatigue on airline flights, which replicates findings from experiments performed in controlled laboratory conditions [40,41]. However, laboratory conditions are not a perfect replication of actual in-flight conditions, and it is important to document these changes in actual flights and in modern aircraft. This is particularly important given that the large number (approximately 2.75 billion passengers fly on commercial airlines worldwide annually [42]) and the average age of air travelers has risen and many more people with chronic diseases fly. Wearables combined with subjective and objective measures open the possibility to study a much broader range of people under real-time flight conditions and provide monitoring of the effects of reduced air pressure on individual symptoms.

Second, we found a strong association between high HR and skin temperature measurements and elevated hs-CRP levels, consistent with previous studies using nonportable devices [43]. For the 603 d of Participant #1 monitoring, elevated hs-CRP, HR, and skin temperature were evident during four periods. Interestingly, for at least one of these periods (Day 518) the participant was clinically asymptomatic, indicating that the inflammatory events were detectable by both a medical hs-CRP test and wearable devices, but not by the participant. Outside of these four periods, at low resolution, we did not detect any days with elevated hs-CRP that did not have elevated HR or skin temperature. Our high-resolution method using only HR also identifies the ill periods as well as four additional peaks and identifies the initial onset of disease for three of the four periods. For three other individuals an elevated HR was detectable during periods of high hs-CRP and illness, and skin temperature was elevated for one individual. In each case, the COH method identified an early stage of the disease. We suggest that wearable devices may be a sensitive measure for detecting certain inflammatory responses, and that in some circumstances, these may even be better than participant-reported observations.

It is possible that the use of wearables will lead to false alarms and overdiagnosis of disease. The number of false alarms will depend upon the threshold that is set, which can be personalized. It is notable that the most severe infection in this study, Lyme disease, which required physician intervention, had strong and repeated COH signals and is readily detected. Overall, we envision that these devices could be particularly powerful for individuals who are responsible for the health of others (i.e., parents and caregivers), and perhaps also for those who have historically limited health care access, including groups with low income and/or remote geography.

Of particular note was the detection of elevated skin temperature and HR as well as the decrease in SpO_2_ at the onset of symptoms of Lyme disease. This information was quite valuable in early diagnosis and treatment and occurred in an instance in which the characteristic “bull’s eye” rash was not observed after initial infection. Indeed, the symptoms first appeared after entry into a country in which Lyme disease was infrequent and the physician's initial recommendation of penicillin may have been an inadequate treatment. Moreover, the detection by wearables was quite robust, as outlying HR and skin temperature measurements were evident at every day of the disease. It is expected that the use of wearables for disease detection is extensible to other individuals and diseases associated with inflammation; obviously, the more serious the disease and associated inflammation, the more likely it will be detected using the portable devices. Indeed, the devices could be set to identify periods of highest inflammation (i.e., the ones that might require physician intervention) in order to reduce false alarms or avoid minor illness not requiring medical intervention.

Elevated HR is a strong predictor of cardiovascular disease and metabolic syndrome and is also associated with IR, insulin precursor presence, and the acute insulin response [44–48]. Hyperinsulinemia can trigger an increase in sympathetic activity through peripheral and central mechanisms [49–51]. Although the feedback loops are complex, this increase in sympathetic activity may contribute to the pathophysiology of IR, hypertension, and cardiovascular disease [52]. Our findings are notable in that we found a strong positive association between the difference of daytime and nighttime HR and participant SSPG levels that was independent of the effect of activity and BMI. This may indicate increased sympathetic activity reflective of complex physiological changes that are associated with IR and progression to diabetes. The fact that these differences can be measured using wearable devices raises the likelihood that this approach may someday be a useful measure for early detection of IR and risk for T2D.

Although many of the observations were originally discovered on a single person (who was also an author in this study—a potential limitation), in all cases, the results were validated on a larger cohort, demonstrating that our results can be generally applied. We also note that although up to seven devices were used simultaneously by a single person in this study, in addition to a scale to measure weight, in principle, all of the parameters measured in this study can be readily captured using two devices, a smartphone and a smartwatch, thus facilitating data collection and integration of diverse data types. Finally, we note that many more analyses of these data can be carried out, some of which are best performed using data collected from individuals operating in a more controlled setting.

In conclusion, this study demonstrates that a diverse array of measurements can be systematically obtained using portable devices and used to monitor health-related physiology and activities. These measurements are likely to be important not only in basic science research but also in a clinical setting. It is likely in the future that these devices will be used by physicians to help assess health states and guide recommendations and treatments [53,54].

## Materials and Methods

### Human Cohorts and Ethics Statement

The participants were enrolled in this study under the IRB protocols IRB-23602 and IRB-34907 at Stanford University; the IRB approved the study and consent forms that were used. All participants consented in writing. All clinical measurements were covered by IRB-23602, the enrollment criteria of which was 18 y of age or older. All the wearable measurements were covered by IRB-34907, with the enrollment criteria as age 13 or older. The 43 activity participants were recruited with efforts to enroll those at risk for T2D (SSPG >140 mg/dL; fasting plasma glucose >100 mg/dL, Oral Glucose Tolerance Test >140 mg/dL, Hemoglobin A1C >5.6%) along with healthy controls. Other than IR and/or moderate hyperglycemia, all participants enrolled in this study are self-reported healthy. Age, sex, and ethnicity information was available for all SpO_2_ participants and 38 of 43 Basis device participants and is indicated in S1 Table.

### Selection of Wearable Devices and Data Acquisition

After evaluating more than 400 available wearable devices at the beginning of the study, we selected several for participants to use. The criteria for selection was (1) ability to access the raw data from the manufacturer, (2) cost, (3) overlap in measurement of at least one component with another device to assist in reproducibility, and (4) ease of use. Participant #1 wore seven portable devices for large segments of this study (Fig 1B); the remainder used a Basis device. For the SpO_2_ measurements, three devices (Scanadu, iHealth-finger, Masimo) were used by Participant #1; either iHealth-finger or Masimo were used by the other participants.

For the Basis data, the manufacturer securely uploaded the data to a secured cloud storage system. For other devices, the data were collected by the user’s smart phone, where the user securely transmitted the data to our repositories.

### Data Description (Devices/Apps)

Each manufacturer and device outputs data in a unique, device-specific format. There are currently no standards and/or best practice recommendations on how the data should be recorded. Below are the data and metrics that were stored.

- Date/Time—all data points were recorded in different formats and annotated to note the time zone of the recording. (MM/DD/YYYY h:min:s -UTC)
- HR—(Basis B1, Basis Peak, Scanadu): BPM
- Accelerometer—(Basis B1, Basis Peak): data are derived on the device as the square root of triaxial acceleration: sqrt(x^2+y^2+z^2)
- Steps—(Basis B1 and Basis Peak, MOVES: algorithmically derived by manufacturer
- Activity—(Basis B1 and Basis Peak, MOVES): categories are algorithmically derived by manufacturer: running, walking, biking, transport (MOVES)
- Skin Temperature—(Basis B1 and Basis Peak), degrees Fahrenheit
- Calories—(Basis B1 and Basis Peak): algorithmically derived by manufacturer

Additional parameters (galvanic skin response, food logging, and continuous glucose metrics) were also collected and will be the subject of another study.

For Participant #1, over 250,000 measurements were recorded daily using a combination of the MOVES App, the Basis device, and other wearables.

### Assessment of the Validity of the Wearables Measurements

Many of the devices have been validated for accuracy by the manufacturer (e.g., Basis: https://258b1w36g2mmq40rp2i2rutg-wpengine.netdna-ssl.com/wp-content/uploads/2015/12/12212015_UCSF_WhitePages.pdf; http://www.mybasis.com/wp-content/uploads/2014/04/Validation-of-Basis-Science-Advanced-Sleep-Analysis.pdf). Nonetheless, we compared the SpO_2_ and HR measurements from the Masimo, Scanadu, iHealth-finger and Basis devices to those from our standard WA 6000 series vital signs monitor used in the clinical service laboratory at Stanford University. Measurements were taken at three or more different days. Finger measurements were made on either the right index finger or right ring (fourth proximal) finger; no detectable differences (<1%) were found when compared to the WA instrument using simultaneous measurements of the WA and wearable device, i.e., simultaneous tests were run on the wearable and WA device; as controls, finger locations were also swapped for the two devices and the WA instrument). To cover a wide range of SpO_2_ levels and HRs, the participant held his breath and the measurements were made simultaneously (within 2 s) on two different locations using one device and the standard instrument. For finger-based measurements, similar numbers of measurements were made switching the device locations. The comparison across devices was done by matching the time stamps. The Bland–Altman method [16,17] and Pearson correlation were applied to assess the agreement and the relationship between the wearables and the clinic devices, respectively. As shown in S1 Fig, for SpO_2_, 100% of the Masimo and iHealth-finger and 85% of the Scanadu measurements were within three percentage points of the WA instrument. For HR, over 95% of all portable device measurements were within three BPM of the clinical equipment (100%, 97.1%, 97.5%, and 93.5% for the Scanadu, iHealth-finger, Masimo, and Basis, respectively.) Although this percentage was slightly lower for the Basis, 100% of the Basis measurements were within the accuracy criteria of the Association for the Advancement of Medical Instrumentation for HR meters (five BPM and ±10% of the WA instrument) [55]. There was no evidence of systematic bias in the measurements (S1A–S1D Fig) with the exception of the Scanadu SpO_2_ measurements, in which the majority of readings were slightly higher than the clinical device and a few were much lower (S1C Fig); in all HR cases, the averages were within one BPM of the mean.

Pearson correlation analyses also revealed tight correlation of the wearables measurements with standard medical devices (R = 0.77 to 0.96, *p* < 0.0005; S1 Fig). The only exception was the Scanadu SpO_2_ measurements (R = 0.46). The Scanadu measures HR and SpO_2_ from the forehead, whereas all the other devices including the standard medical device record measurements from the finger. It is not clear whether our findings are due to technical differences of the device or the location of measurement [56]. Regardless, as described below, the trends for SpO_2_ levels (and other parameters) under different conditions are identical among each of the different devices.

In addition to the physiological measurements, we also assessed the accuracy of the activity data. First, we examined the agreement and correlation between the activity-sensing devices (Basis, MOVES, Withings) (See S1 Fig). The Pearson correlations between devices ranged from 0.74 to 0.81 (all *p*-values <0.00001; S1 Fig). The Bland–Altman Plots revealed that at daily step counts less than 12,000 steps, there was good agreement between the Basis and Withings devices. However, as daily step counts increased above 15,000 steps, the Basis gave higher step counts than the Withings device. Both Basis and Withings devices gave higher measurements than the MOVES device. The MOVES step measurement was compared using absolute measurements. Specifically, we manually counted 100 steps 12 separate times at three different locations (Bay area, Geneva, Uppsala) and compared the MOVES-recorded steps with the actual steps. The values recorded were found to be 0.79 +/- 0.16 SD of the actual value. To measure running distances, we compared two runs over a measured distance of 3.2 miles; the measured values were 0.94 +/- 0.04 that of the actual value. We also performed similar comparisons at different geographical locations by analyzing 13 outdoor runs at three different locations and compared the distances with those derived from Google Maps (for two locations, distance was confirmed using an automotive odometer). The values recorded were found to be 0.96 +/- 0.05 SD to that measured using Google Maps. Comparison of MOVES results with those of three runs using treadmills showed a larger difference of 0.75 +/- 0.22 SD.

To assess the agreement of measurements of steps using the Basis Peak device and MOVES and Withings applications, we compared the total number of steps per day for the 132 “nontravel days.” Time zone conversion was applied to make the three devices comparable. The Bland–Altman method and Pearson correlation were applied to assess the agreement and the relationship between the devices (S1 Fig).

We note that the devices were assessed under a limited set of conditions and that not all possible conditions were assessed.

### Analysis of Circadian and Diurnal Rhythms in Physiological Parameters

To explore the 24-h distribution of physiological parameters, we focused on 71 “nontravel days” by excluding the days when a time zone other than the home time zone was reported by the MOVES GPS parameter. To eliminate the possible effect from jetlag, we removed the entire last traveling day and also the following 2 d after travelling. (We note the results were very similar to those when no extra days were removed, indicating that the effect of jetlag on the patterns shown in Fig 2 are small (not shown)). The mean of the physiological parameters (measured by Basis Peak) for each hour per day were reported in the heatmap Figure (S2 Fig), and the overall hourly distribution of the 71 d was summarized in box plots (Fig 2). The sleep time per hour was defined as the percentage of times designated as sleep (Basis Peak) compared to the total number of hours (71 d) in each hour window. Either the standard time or the daylight saving time was selected in the analysis, depending on the time of the year.

### Physiological Response to Different Human Activities

We binned the Basis measurements (HR, skin temperature, steps, and calories) into different activity categories (walking, running, cycling, sleep) according to the information from Basis and the MOVES app and compared the distribution in each bin (S3 Fig).

### Flight Tracking and Cohort Information

Flight information was obtained using FlightAware (https://flightaware.com/). Flight information was accessed using the FlightXML API using Python SOAP client library Suds. For Participant #1, exact take-off and landing times (within 1 min) were recorded for >95% of flights. For one out of the first 20 flights, SpO_2_ was measured by Masimo device only at the cruise stage, and this is the flight that does not show inverse correlation between SpO_2_ measurements and altitude.

Eighteen individuals participated in the flight study, and their age, ethnic background, and gender information are summarized in S1B Table. Participant #1 used Masimo, Scanadu Scout, and iHealth-finger device; Participants #16 and #44–#46 used iHealth-finger device; Participants #20, #47–#57, and #60 used Masimo device. Fig 4D shows the SpO_2_ distribution for all the participants (shown for Participant #1 were data recorded by iHealth-finger device).

### Assessing the Relationship between Tiredness and SpO_2_ Levels

SpO_2_ levels were measured by either Masimo or Scanadu Scout devices. Meanwhile, Participant #1 logged the status of “tired” and “alert,” and we compared the wearable-measured SpO_2_ levels between the two statuses according to the notes. To be more objective in defining fatigue, the participant also performed psychomotor vigilance test (Canadian tiredness test) (http://www.painfreesleep.ca/tiredness-test?&cid=semeOyQHbZq) in two separates flights besides the self-reported system. Specifically, this test evaluates the participant’s fatigue status by measuring the participant’s response time to a visual stimulus. For each measurement, response time to 12 visual stimuli were measured. Missed signal with response time slower than 500 milliseconds was counted as 500 milliseconds in the calculation.

### Oral Temperature Measurement

The oral temperature of Participant #1 during Days 471–474 (Lyme disease infection) was measured by an oral thermometer (Day 471 8:00 a.m.: 100.7°F; Day 471 7:00 p.m.: 100.2°F; Day 472 8:00 a.m.: 98.9°F; Day 473 11:00 a.m.: 100.7°F; Day 474 3:00 p.m.: 102°F; Day 474 6:00 p.m.: 101.4°F).

### Diagnosis of Diseases using Wearables-Measured Physiological Parameters

To investigate the ability of wearables to predict and monitor disease, a normalization framework was developed to accommodate the dynamic change caused by different activities and make measurements comparable. To normalize resting Basis-measured HR and skin temperature, we first excluded all the measurements recorded during or immediately after exercise that usually generate large variation in physiological parameters. Specifically, all records used have step measurements of zero for at least 10 min previous to that time point (including the current minute) and are also not associated with any prediction of activity (by MOVES software, if applied), including walking, running, cycling, or flying time (by personal calendar). Data in second resolution were first converted to minute resolution by calculating the median value. After filtering the activity-related data, we further performed Z-transformation (standardize) to the measurements based on the baseline norm of sleep status and nonsleep status (predicted by Basis device). Percentage-of-outliers was defined as percentage of measurements deemed outliers for each day by comparison with the personalized, activity-specific (sleep and nonsleep at resting) mean for the overall monitoring period (the baseline value; Z-Score >2). For Participant #58, whose sleep data are missing, the data were normalized based on the personalized 24-h distribution. Data from Basis B1 and Basis Peak were normalized separately to minimize the difference between devices. Overall, a period of 679 d (from Day 63 to Day 741) was examined. In this period, Basis data were missing for 76 d, therefore the analysis was performed on the remaining 603 d in this period. To capture data on both travel and nontravel days, we defined the start and end of a day according to coordinated Universal Time, which is 7 or 8 h ahead of Pacific Daylight Time or Pacific Standard Time.

For the Day 470 flight, we assessed the Scanadu-measured SpO_2_ readings (flight duration time = 94 min) relative to other flights by collecting all of the SpO_2_ readings recorded by Scanadu during flights with similar flight time (duration time <120 min). The readings in each of the five flight stages were compared separately and the significance of the difference was assessed by two-sided Wilcoxon rank sum test.

For the analysis of illness with daily resolution, we detected abnormally elevated HR and skin temperature during the four periods reported. Specifically, we detected abnormally elevated HR (ranks #8, #2, #16, #3, and #1 out of 603 d, respectively) and skin temperature (ranks #1, #4, #10, #2, and #5 out of 603 d, respectively) for the period from Days 470–474; we detected abnormally elevated HR (ranks #7 out of 603 d) and skin temperature (ranks #12 out of 603 d) for Day 518; we detected abnormally elevated HR (Day 455 ranks #12 out of 603 d) and skin temperature (Day 456 ranks #6 out of 603 d) for the period from Days 455–456; we detected abnormally elevated HR (Day 667 ranks #4 out of 603 d) and skin temperature (Day 667 ranks #24 out of 603 d) for the period from Day 665 to Day 669.

To map inflammatory disease at higher resolution, we further analyzed the normalized HR. Specifically, we first smoothed the normalized HR using a moving average filter and then applied peak detection to identify local maxima of the smoothed signal. We used “smooth” and “findpeaks” packages in matlab to perform smooth and peak finding. To identify isolated peaks different from the global and local distribution at high confidence, we set “MinPeakHeight” to equal to two, “MinPeakDistance” to equal to “span” (3-h), and “MinPeakProminence” to equal to two. The optimized hyper-parameter “span” (3-h) was selected by training the model on Participant #1 and was applied when analyzing other individuals.

To evaluate the predictive power of the method in distinguishing the sick periods from the healthy periods, we defined a set of sick periods (positive set) based on self-reported symptoms and the relevant blood test. In the positive sets, we also included 3 d before the day when the symptom was reported or evidenced by blood work to acknowledge the fact that abnormal physiological signal might occur before the self-reported symptom. As a negative control, we followed the same rule and defined a set of periods either (1) composed of the days with normal CRP measurements or (2) composed of all days during the monitoring periods that are not included in the positive sets. We used binary scoring of each event by the presence or absence of the peak in the period. Each sick period was counted as one event. The area under the ROC curve was calculated to evaluate the classification power. We also employed cross-validation procedures to avoid overfitting to Participant #1’s data.

### Personalized Physiological and Activity Profiles for 43 Individuals

Of the 43 individuals tracked using Basis devices, 28 wore only Basis B1 devices, 9 wore only Basis Peak devices, and 6 wore both the Basis B1 and the Basis Peak. The Basis Peak has improved HR sensing during exercise as compared to the Basis B1; the resting HR and other parameters were comparable between the two devices. For the cohort-level analyses, 17.1 mo of Participant #1’s data were used. We used activity normalization as well as device-specific normalization, as described below, to account for potential differences between the two devices. For each of the 43 individuals, we calculated average biometric values for HR, skin temperatures, and activity. For HR and skin temperature, we used measurements occurring at time points at which there were zero steps recorded at the current time point as well zero steps recorded within the 10 s previous to that time point. These periods corresponded to activity designations of inactive, light activity, and sleep by the manufacturer’s algorithms.

The number of days recorded for each individual was calculated as the difference between the date the recording began and the date the recording ended or the date on which the data were accessed, whichever came first. We calculated the average number of steps per day for each of the 43 individuals by multiplying the average number of steps per second by the number of seconds in a day (86,400 s).

To capture daytime versus nighttime biometrics, we restricted our measurement capture window to 1 h during the day (3–4 p.m.) when our participants were awake and had taken more than 30 steps during this hour to guarantee a minimal level of activity, and compared this to 1 h during the nighttime (3–4 a.m.) when our participants appeared to be asleep and inactive with a threshold of less than five steps during this hour to ensure inactivity during sleep, but allowing for minimal measurement artifacts or limb movement during sleep.

Daily activity habit plots were created for each individual by generating smooth conditional mean lines with a 95% CI of accelerometer magnitude data by hour of day using generalized additive models (ggplot2 geom_smooth in R). Individuals were classified into one of four groups based on the peak characteristics of the curve. To automate this process, functional clustering using the R package FClust [57] was done on the activity curves to cluster members by similarity of activity curve characteristics (S2 Fig).

### Comparison with Clinical Information

For the cohort that was monitored by the Basis devices, a subset of our participants had standard clinical panels (e.g., fasting plasma glucose, glycated hemoglobin [HbAlc], blood cell counts, etc.; S3 Table; performed in the Stanford clinical labs) and demographic information. The data were accessed using the Stanford Translational Research Integrated Database Environment (STRIDE) [58]. Thirty-eight participants with Basis datasets were annotated for gender (18 male and 20 female) and baseline BMI.

Twenty participants had undergone the modified insulin suppression test after an overnight fast (48). The test consisted of a 180-min octreotide (0.27μg/m^2^/min), insulin (0.25 μg/m^2^/min), and glucose (240 μg/m^2^/min) infusion with blood draws at minutes 150, 160, 170, and 180. Blood glucose was measured using the oximetric method, and the SSPG is the mean of the four measurements [12,13,59]. IR is defined as a SSPG ≥140 mg/dL (*n* = 12), and insulin sensitivity is defined as <140 mg/dL (*n* = 8).

We analyzed average HR and skin temperature values for men and women using the 38 Basis datasets using an unpaired, two-tailed two-sample *t* test with Welch correction for potential unequal variation in the two populations. Pearson correlation between the average number of steps per day and average resting HR, as well as average number of steps per day and delta BMI (year 0 [baseline] minus year 1 BMI measurements) were done using R. The evaluation of the association between steps, HR (daytime, nighttime, and difference between day and night), and SSPG was done using SAS 9.4^®^ (SAS Institute, Inc., Cary, NC. 2013). To account for unequal variances, we used a restricted maximum likelihood approach with a robust variance estimator to estimate the regression coefficients and their 95% CIs.

## Supporting Information

S1 Fig Accuracy of the devices. Bland Altman plots of the level of agreement between the Welch Allen clinical device and wearable sensors for SpO_2_ (A) and heart rate (B). The difference histograms for SpO_2_ (C) and heart rate (D) show percentage of measurements by level of difference between each of the wearable sensor devices and the clinical device. The SpO_2_ number of measures per device is the same as those given by device for heart rate (D) with the exception of the Masimo device (n = 67 SpO_2_). The Pearson correlation plots (E) show the degree of correlation between the wearable sensor devices and the clinical device. Bland-Altman (F), Difference histogram (G) and Pearson correlation (H) plots of pair-wise comparison of step measurements between MOVES, Basis and Withings. In the Bland-Altman plots, the y-axis is the difference between both measurements and the x-axis is the average of the two measurements. The red line is the mean difference; the purple line and green lines are the upper and lower 95% limits of agreement respectively. The histograms provide a more quantitative measure of bias. In the Pearson correlation plots (E, H) symbol color represents number of overlapped points (blue: small number; magenta: large number). Measurements were done on a single individual (Participant #1).
(PDF)

S2 Fig Circadian and Diurnal patterns in physiological parameters. (A-B) Heat map showing circadian changes in heart rates (A) and skin temperature (B) as measured using the Basis Peak device over 71 non-travel days. Measurements were done on Participant #1. The heat map of heart rates was organized by weekdays (A). (C) Four general daily activity patterns of the 43 study participants plotted according to actual values at the indicated times. (D) Functional Clustering (k = 4 groups) was done on the activity curves to automate the method of clustering members by the similarity of activity curve characteristics.
(TIFF)

S3 Fig Physiological parameters change dynamically with human activity. Box plot shows dynamic changes of Basis-measured physiological parameters (A: HR, B: Steps, C: Calories, D: Skin temp) with different activities (Sleep category designated by Basis; walking, cycling, running categories designated by MOVES app). Data were collected on Participant #1.
(TIFF)

S4 Fig Correlations between parameters measured by the Basis device. (A) Correlation between HR and Activity. The y-axis values are the mean of HR for each Activity ventile. The ventiles were binned based on accelerometer data for Activity. (B, C) Difference in mean HR (B) and Skin Temperature (C) between the highest decile and lowest quartile of Activity data, binned by accelerometer. The highest decile was used to capture higher impact activity (High Activity) while the lower quartile was used to capture low impact activity (Low Activity). Color shade represents the overall activity levels for each individual, with darker colors corresponding to the highest number of steps per day and lighter colors corresponding to fewer steps per day, ranging from 594 to 10,858 step/day for all individuals.
(TIFF)

S5 Fig Investigating SpO_2_ measurements during flight. (A) Example of SpO_2_ measurements taken by Scanadu (blue) and iHealth-finger (red) on a typical flight. (B) Summary of distribution of SpO_2_ values at different flight stages measured using the iHealth-finger device. (C) Box plot of the distribution of Scanadu-measured SpO_2_ readings classified as “tired” or “work” from non-flying moments (left panel) and “tired” or “non-tired” at the cruise flight stage (right panel). Significance of differences was assessed using two-sided Wilcoxon rank sum test. (D) Scatter plot of response time and SpO_2_ level recorded during one flight. The data recorded during another flight was shown in Fig 4F. Here, response time was derived from psychomotor vigilance test to objectively quantitate the tiredness of the subject. Cyan triangles, purple squares and red dots represent self-reported ‘tired’, ‘in-between’ and ‘alert’ status, respectively. (E) (Upper panel) Median SpO_2_ level measured at the last quarter of the flight (yellow bars) and one of the other three quarters (blue bars). (Lower panel) Durations time of the flights. (F) CDF plot of Scanadu-measured SpO_2_ levels >7hr after takeoff (red) vs. <2hr after take off (blue). All the data points were recorded at the altitude larger than 35000 ft. Significance of the difference between the two distributions was assessed by two sample Kolmogorov-Smirnov test).
(TIFF)

S6 Fig Relationship between flight altitude and SpO_2_ measurement. Plot showing aggregate data from all flights (A) or short flights (B) with Masimo records for Participant #1. (C-G) Data from individual long flight Participant #1 took (only those with the complete record are shown). Symbol color indicates the time after departure. (H) Plot showing the relationship between the maximum altitude and the delta median SpO_2_ for all participants with all flights. The delta median SpO_2_ was calculated as the difference in median SpO_2_ between the maximum altitude and at the ground. Each symbol represents a flight with Mamiso records. Multiple flights from one participate were shown by the same symbol with the same color. At personal level, a significant correlation was observed between the maximum altitude and the delta SpO_2_ value for the two individuals with more than four flights (r = -0.52, *P*-value < 0.002; r = -0.86, *P*-value < 0.004, respectively). (I) As for *H*, but focusing on short flights. (r = -0.71, *P*-value < 0.004; r = -0.96, *P*-value < 0.0002, respectively).
(TIFF)

S7 Fig Using wearables to assist disease diagnosis. (A) 679 day monitoring period for Participant #1. Left: elevated CRP periods; Right: fraction of outlying resting HR and skin temperatures (see Material and Methods). (B-C) Scatter plot of oral temperature and heart rate (B) and skin temperature (C) measured during the Lyme disease period. (D) Results from Lyme disease Antibody blot on Day 487. (E) CRP measurements are plotted against the proportion of daily skin temperature measurements that were more than two standard deviations above the mean for Participant #59 (F) CRP measurements are plotted against the proportion of daily heart rate measurements that were more than two standard deviations above the mean for Participant #37. (G) The timelines for the illness progression, CRP measurements, and Basis monitoring period captured in the figure are indicated for Participant #37. (H) Normalized HR at sick periods in minute resolution for Participant #37. Red peak: Abnormal periods indicated by the peak caller. Red vertical line: CRP larger than 2.5; Green vertical line: CRP larger than 1 but smaller than 2.5; Yellow vertical line: CRP smaller than 1. No peak was detected before the first CRP test since Participant #37 started to wear Basis device after the test.
(TIFF)

S8 Fig Physiological summary of Participant #1. (A) Plot of fraction of outlying skin temperatures and heart rates for all 679 days of Participant #1. Connection was made by time. Line color indicates the health status (blue: health days; red: viral disease; yellow: high CRP event 1; purple: high CRP event 2; green: Lyme disease). (B) ROC curves showing classification power of the COH method in distinguishing the sick periods from the health periods. For each individual, two ROC curves are shown based on different definition of the negative set: (1) negative set was defined as days with normal CRP measurements (Participants #1, #58 and #59 and #37: purple solid line, AUC = 1); (2) negative set includes all the days in the measuring period which are not include in the positive set (Participant #1: red solid line, AUC = 0.983; Participant #58: blue solid line, AUC = 0.960; Participant #59: yellow solid line, AUC = 0.989; Participant #37: purple solid line, AUC = 1).
(TIFF)

S1 Table Characteristics of our cohort. (A) 43 individuals participate in the analysis of personal baseline and health (B) flight study (C) Survey results to determine the location of the probe (inside or outside of the wrist, higher or lower on the arm, in the second column of the table) and whether the probe was always flush with the skin (based on tightness level of watch, on a scale of 1–4, where the descriptors for each numerical value were: 1. Very loose (watch turns around sometimes); 2. Somewhat loose (1–2 fingers fit under band, but no watch turning); 3. Tight (can just barely fit 1 fingertip under band); 4. Very tight (cannot fit any fingertips under band, watch cannot be tightened any further).
(XLSX)

S2 Table Summary of flights analyzed for SpO_2_. (A) Participant #1 (B) Other 17 participants.
(XLSX)

S3 Table Clinic test performed. (XLSX)

## Abbreviations

BMI: body mass index
BPM: beats per minute
COH: Change-of-Heart
HR: heart rate
hs-CRP: high-sensitivity C-reactive protein
IR: insulin resistance
IRB: Institutional Review Board
mRem: millirem
SD: standard deviation
SpO_2_: peripheral capillary oxygen saturation
SSPG: steady-state plasma glucose
T2D: type 2 diabetes
WA: Welch Allyn
