# Supplementary figures and images for: Digital Health: Tracking Physiomes and Activity Using Wearable Biosensors Reveals Useful Health-Related Information
Source: PLoS Biol. 2017 Jan 12;15(1):e2001402. doi: 10.1371/journal.pbio.2001402 (PMC5230763; doi:10.1371/journal.pbio.2001402)

**A**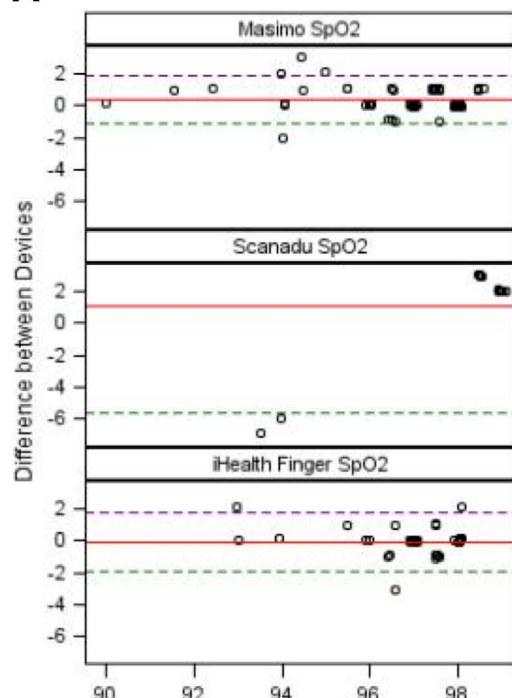**C**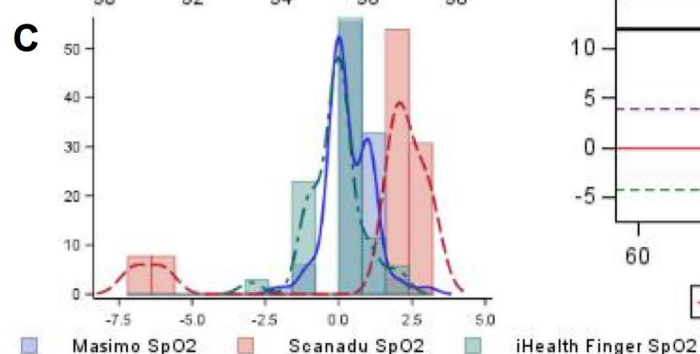**D**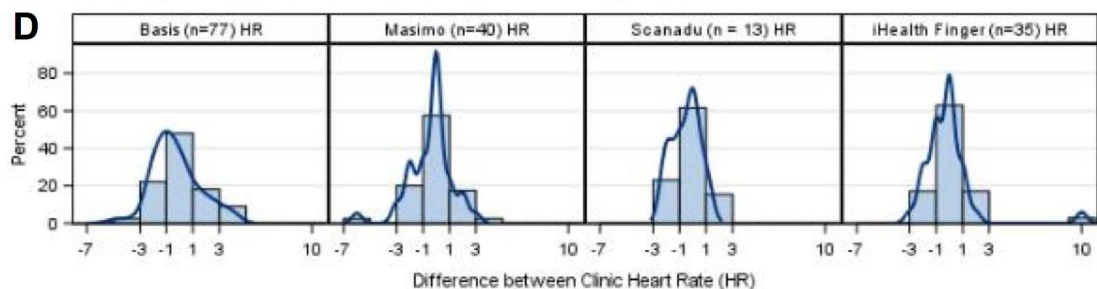**B**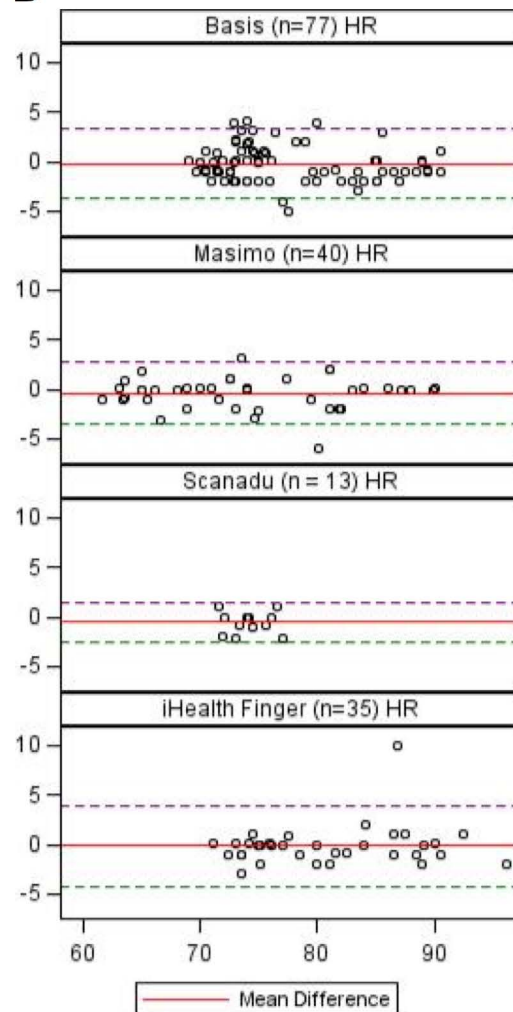**E**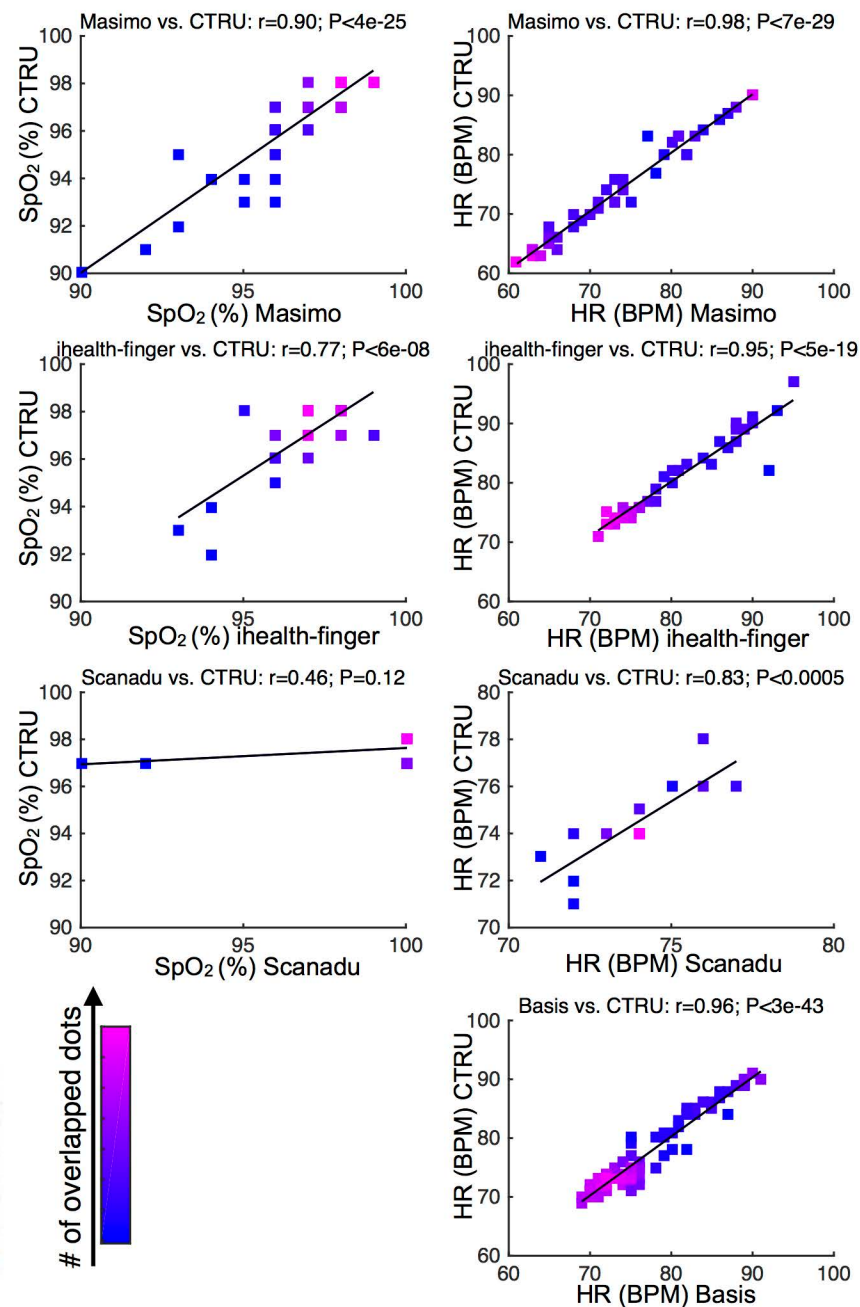

F

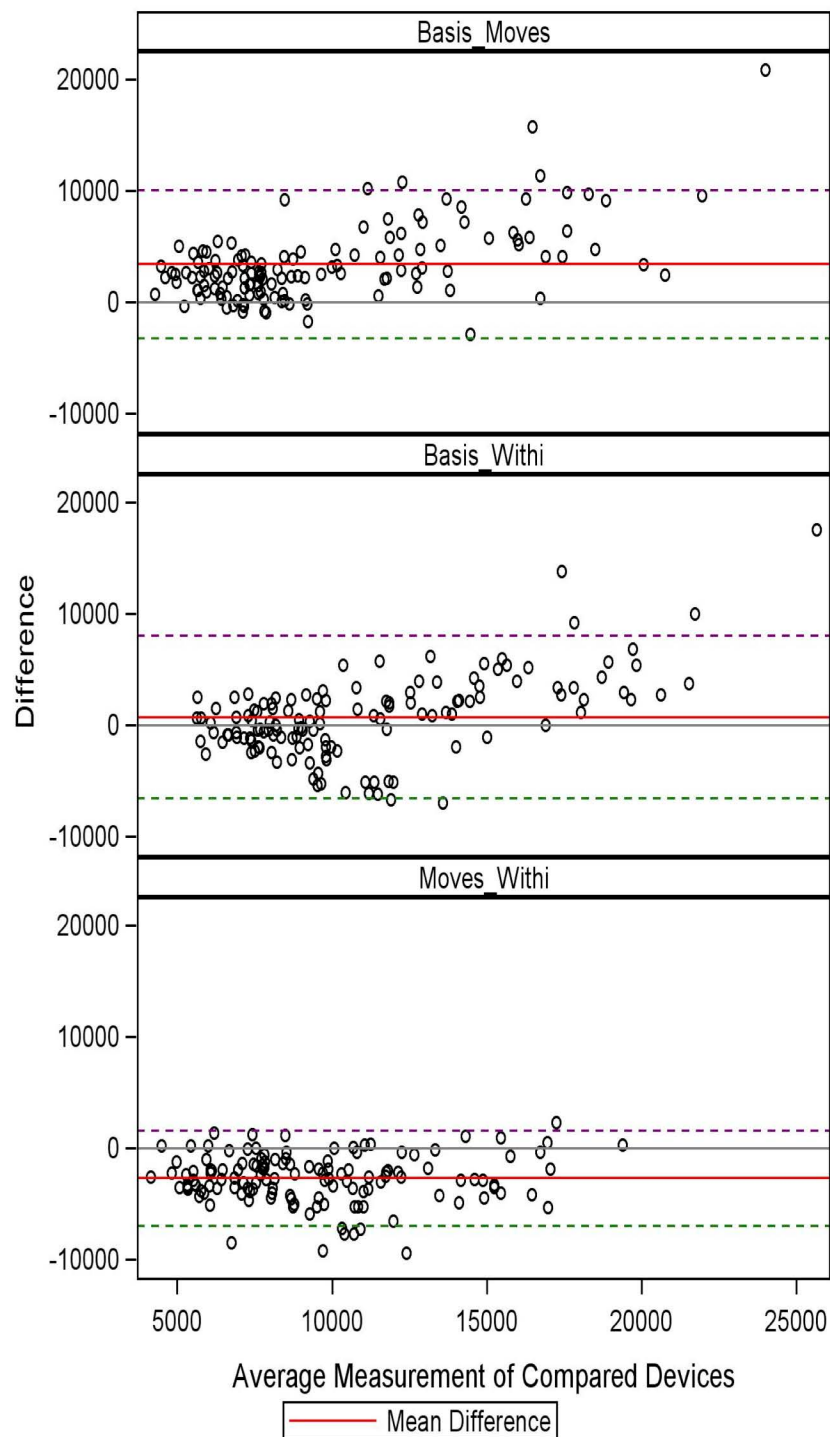

G

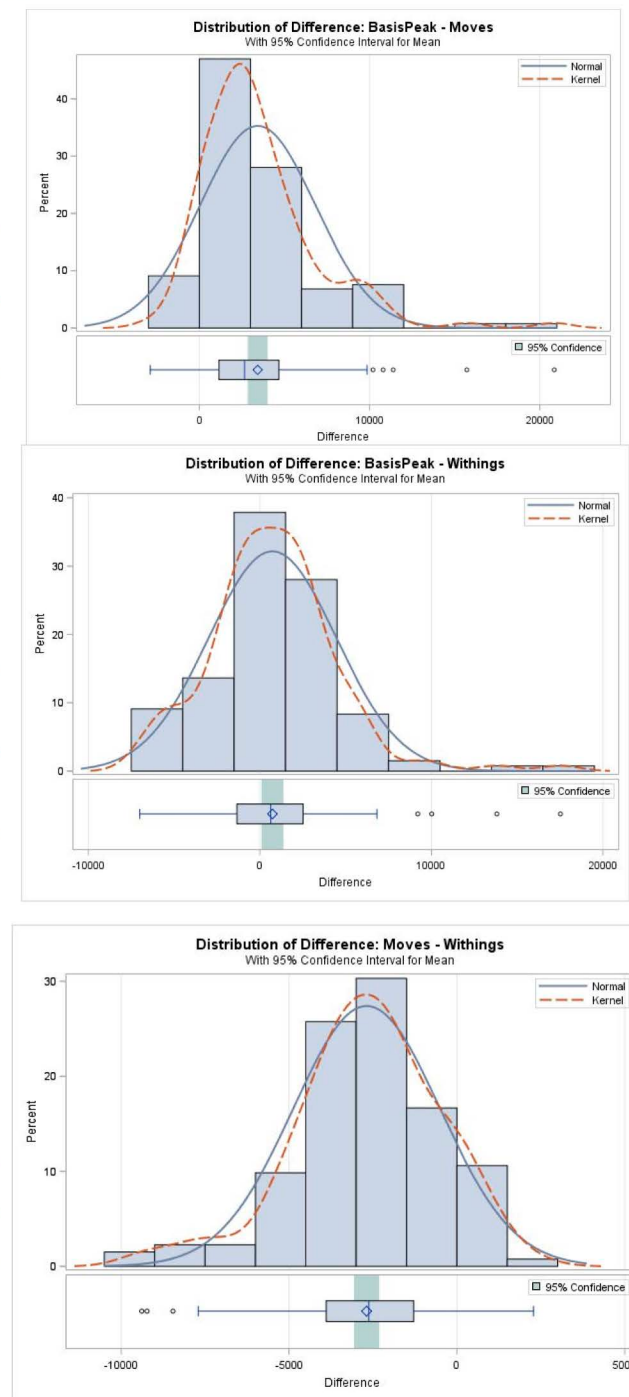

H

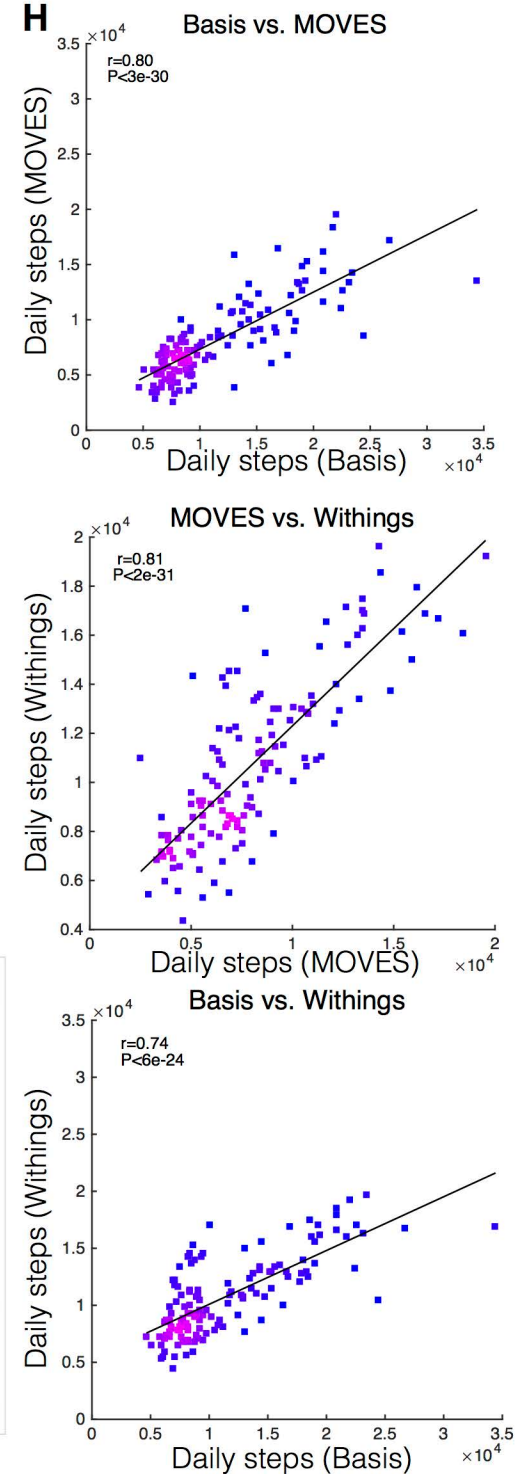

S1 Fig

Supplement: S1 Fig — Bland Altman plots of the level of agreement between the Welch Allen clinical device and wearable sensors for SpO2 (A) and heart rate (B). The difference histograms for SpO2 (C) and heart rate (D) show percentage of measurements by level of difference between each of the wearable sensor devices and the clinical device. The SpO2 number of measures per device is the same as those given by device for heart rate (D) with the exception of the Masimo device (n = 67 SpO2). The Pearson correlation plots (E) show the degree of correlation between the wearable sensor devices and the clinical device. Bland-Altman (F), Difference histogram (G) and Pearson correlation (H) plots of pair-wise comparison of step measurements between MOVES, Basis and Withings. In the Bland-Altman plots, the y-axis is the difference between both measurements and the x-axis is the average of the two measurements. The red line is the mean difference; the purple line and green lines are the upper and lower 95% limits of agreement respectively. The histograms provide a more quantitative measure of bias. In the Pearson correlation plots (E, H) symbol color represents number of overlapped points (blue: small number; magenta: large number). Measurements were done on a single individual (Participant #1). (PDF) [file pbio.2001402.s001.pdf]

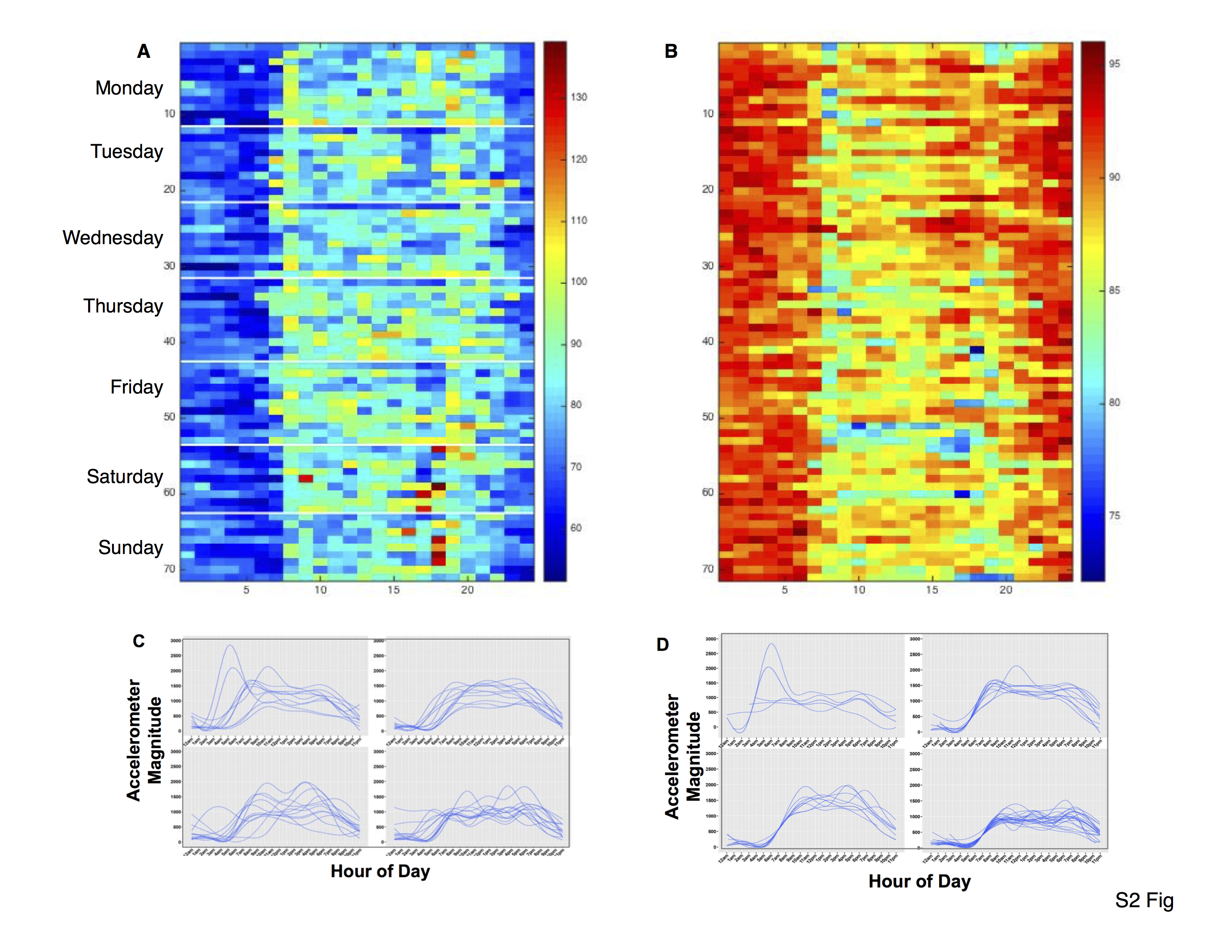

Supplement: S2 Fig — (A-B) Heat map showing circadian changes in heart rates (A) and skin temperature (B) as measured using the Basis Peak device over 71 non-travel days. Measurements were done on Participant #1. The heat map of heart rates was organized by weekdays (A). (C) Four general daily activity patterns of the 43 study participants plotted according to actual values at the indicated times. (D) Functional Clustering (k = 4 groups) was done on the activity curves to automate the method of clustering members by the similarity of activity curve characteristics. (TIFF) [file pbio.2001402.s002.tiff]

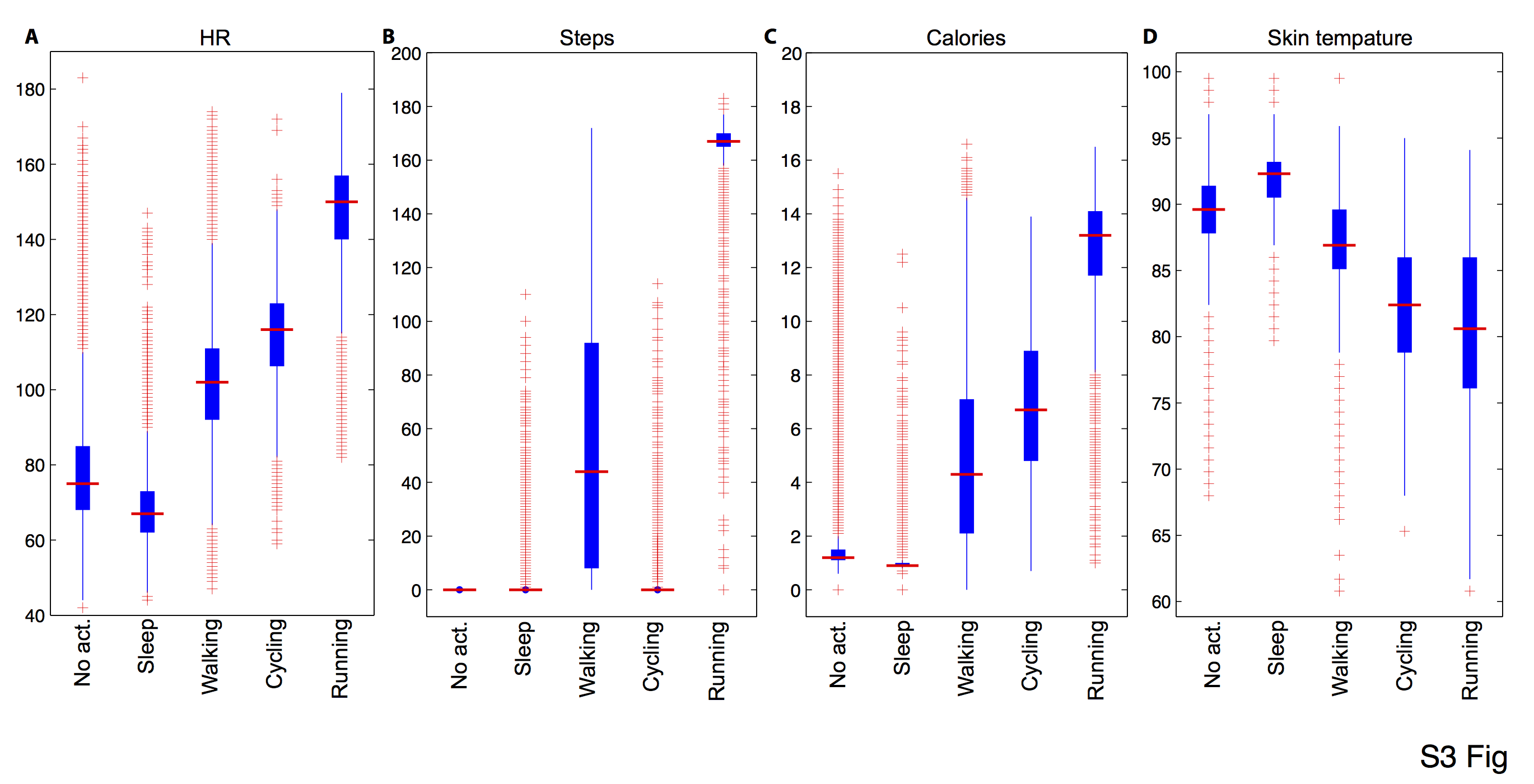

Supplement: S3 Fig — Box plot shows dynamic changes of Basis-measured physiological parameters (A: HR, B: Steps, C: Calories, D: Skin temp) with different activities (Sleep category designated by Basis; walking, cycling, running categories designated by MOVES app). Data were collected on Participant #1. (TIFF) [file pbio.2001402.s003.tiff]

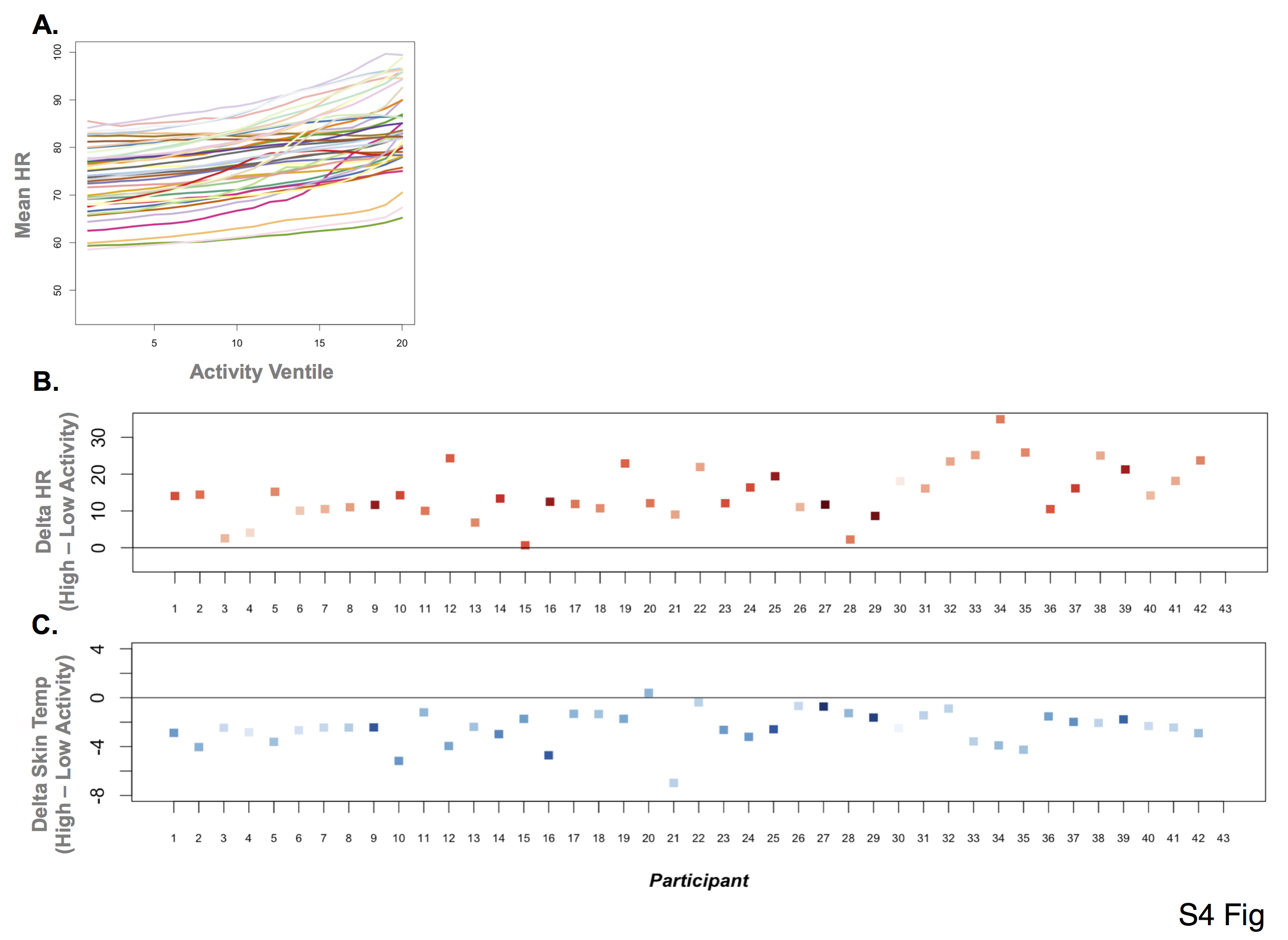

Supplement: S4 Fig — (A) Correlation between HR and Activity. The y-axis values are the mean of HR for each Activity ventile. The ventiles were binned based on accelerometer data for Activity. (B, C) Difference in mean HR (B) and Skin Temperature (C) between the highest decile and lowest quartile of Activity data, binned by accelerometer. The highest decile was used to capture higher impact activity (High Activity) while the lower quartile was used to capture low impact activity (Low Activity). Color shade represents the overall activity levels for each individual, with darker colors corresponding to the highest number of steps per day and lighter colors corresponding to fewer steps per day, ranging from 594 to 10,858 step/day for all individuals. (TIFF) [file pbio.2001402.s004.tiff]

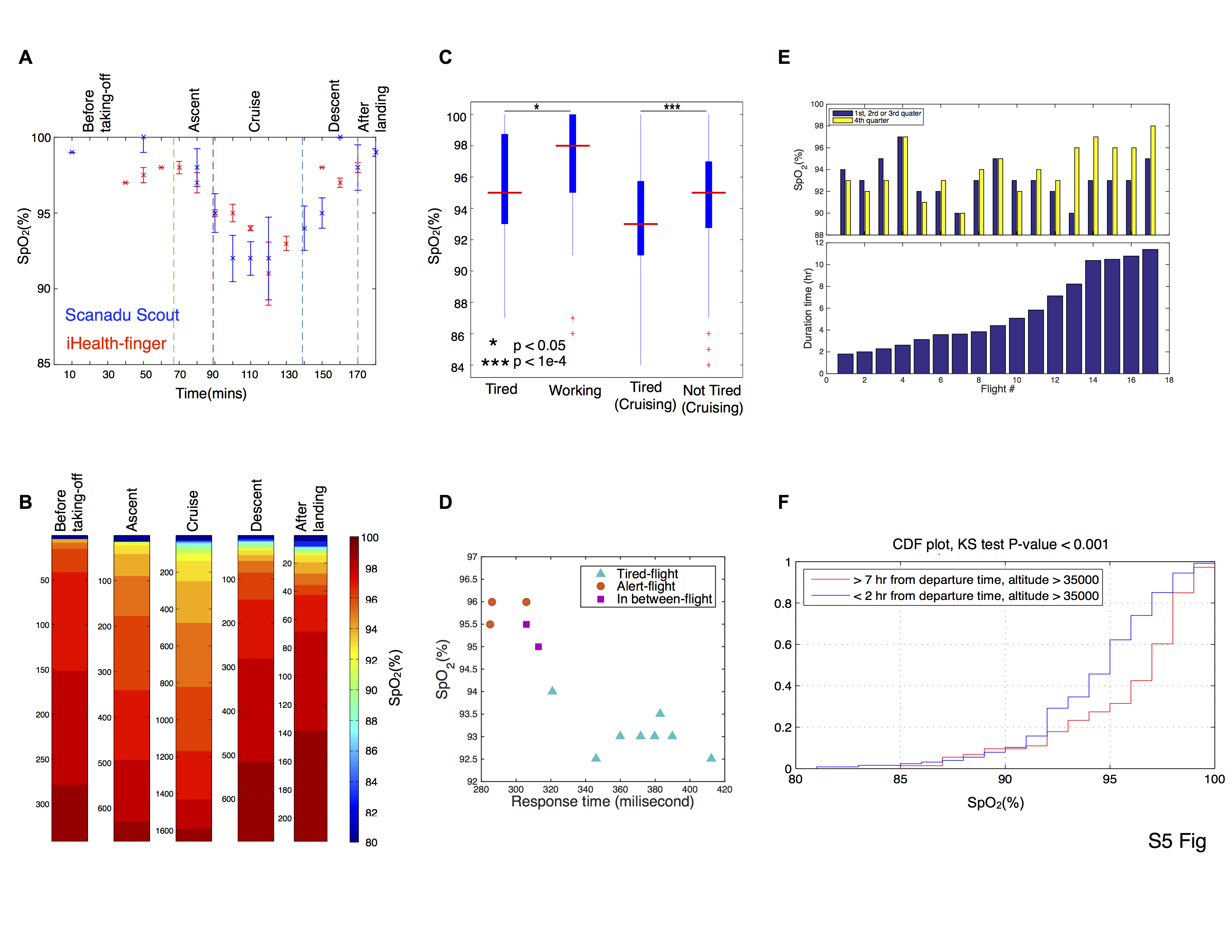

Supplement: S5 Fig — (A) Example of SpO2 measurements taken by Scanadu (blue) and iHealth-finger (red) on a typical flight. (B) Summary of distribution of SpO2 values at different flight stages measured using the iHealth-finger device. (C) Box plot of the distribution of Scanadu-measured SpO2 readings classified as “tired” or “work” from non-flying moments (left panel) and “tired” or “non-tired” at the cruise flight stage (right panel). Significance of differences was assessed using two-sided Wilcoxon rank sum test. (D) Scatter plot of response time and SpO2 level recorded during one flight. The data recorded during another flight was shown in Fig 4F. Here, response time was derived from psychomotor vigilance test to objectively quantitate the tiredness of the subject. Cyan triangles, purple squares and red dots represent self-reported ‘tired’, ‘in-between’ and ‘alert’ status, respectively. (E) (Upper panel) Median SpO2 level measured at the last quarter of the flight (yellow bars) and one of the other three quarters (blue bars). (Lower panel) Durations time of the flights. (F) CDF plot of Scanadu-measured SpO2 levels >7hr after takeoff (red) vs. <2hr after take off (blue). All the data points were recorded at the altitude larger than 35000 ft. Significance of the difference between the two distributions was assessed by two sample Kolmogorov-Smirnov test). (TIFF) [file pbio.2001402.s005.tiff]

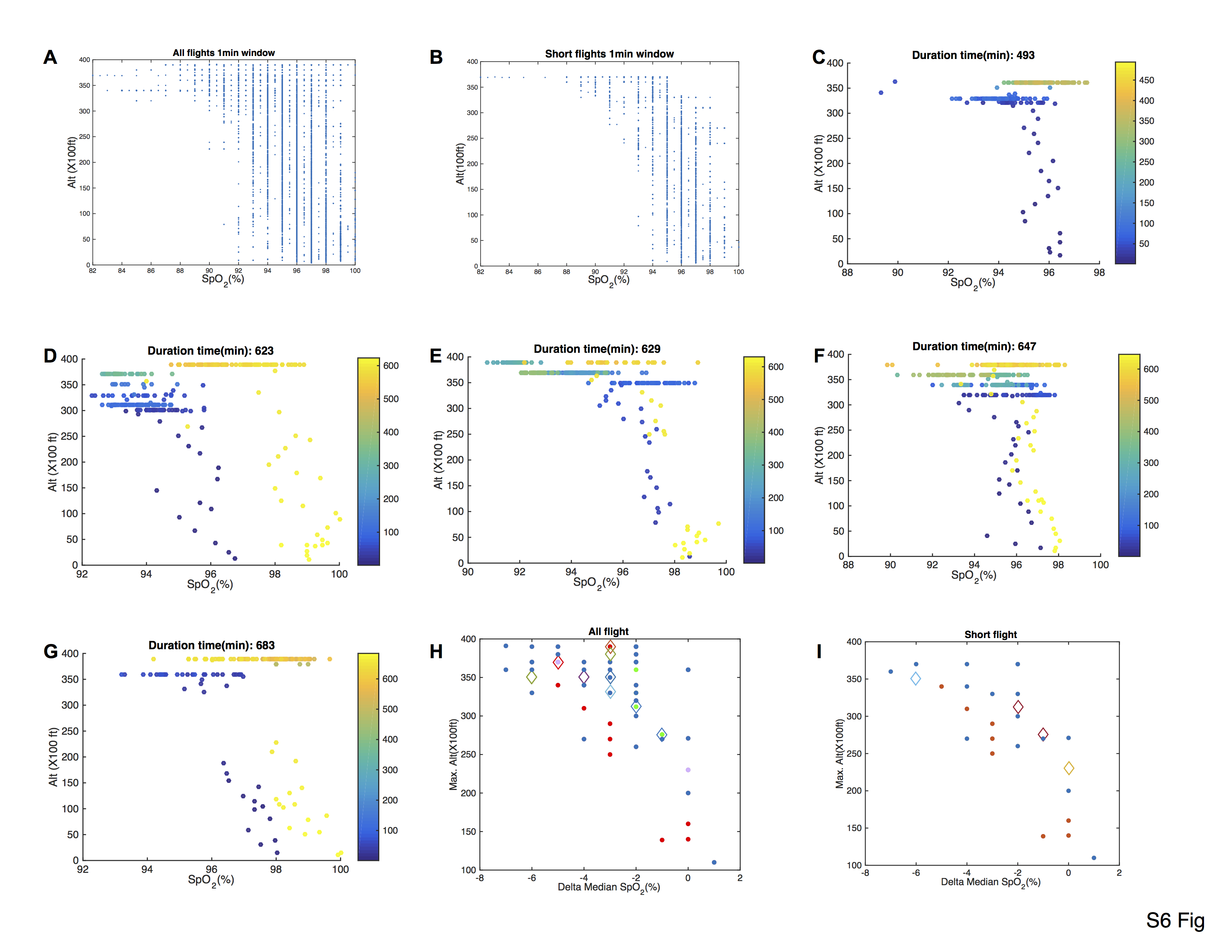

Supplement: S6 Fig — Plot showing aggregate data from all flights (A) or short flights (B) with Masimo records for Participant #1. (C-G) Data from individual long flight Participant #1 took (only those with the complete record are shown). Symbol color indicates the time after departure. (H) Plot showing the relationship between the maximum altitude and the delta median SpO2 for all participants with all flights. The delta median SpO2 was calculated as the difference in median SpO2 between the maximum altitude and at the ground. Each symbol represents a flight with Mamiso records. Multiple flights from one participate were shown by the same symbol with the same color. At personal level, a significant correlation was observed between the maximum altitude and the delta SpO2 value for the two individuals with more than four flights (r = -0.52, P-value < 0.002; r = -0.86, P-value < 0.004, respectively). (I) As for H, but focusing on short flights. (r = -0.71, P-value < 0.004; r = -0.96, P-value < 0.0002, respectively). (TIFF) [file pbio.2001402.s006.tiff]

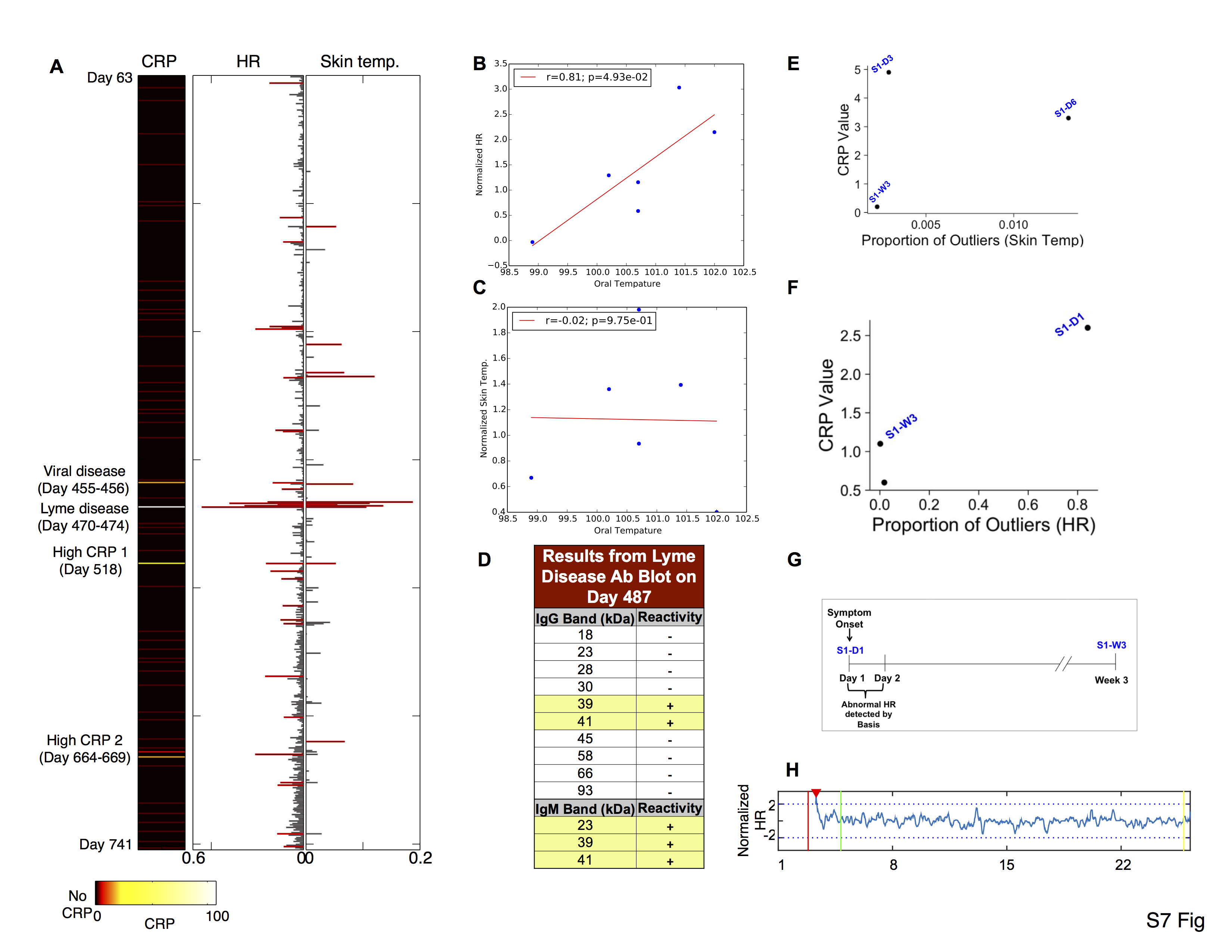

Supplement: S7 Fig — (A) 679 day monitoring period for Participant #1. Left: elevated CRP periods; Right: fraction of outlying resting HR and skin temperatures (see Material and Methods). (B-C) Scatter plot of oral temperature and heart rate (B) and skin temperature (C) measured during the Lyme disease period. (D) Results from Lyme disease Antibody blot on Day 487. (E) CRP measurements are plotted against the proportion of daily skin temperature measurements that were more than two standard deviations above the mean for Participant #59 (F) CRP measurements are plotted against the proportion of daily heart rate measurements that were more than two standard deviations above the mean for Participant #37. (G) The timelines for the illness progression, CRP measurements, and Basis monitoring period captured in the figure are indicated for Participant #37. (H) Normalized HR at sick periods in minute resolution for Participant #37. Red peak: Abnormal periods indicated by the peak caller. Red vertical line: CRP larger than 2.5; Green vertical line: CRP larger than 1 but smaller than 2.5; Yellow vertical line: CRP smaller than 1. No peak was detected before the first CRP test since Participant #37 started to wear Basis device after the test. (TIFF) [file pbio.2001402.s007.tiff]

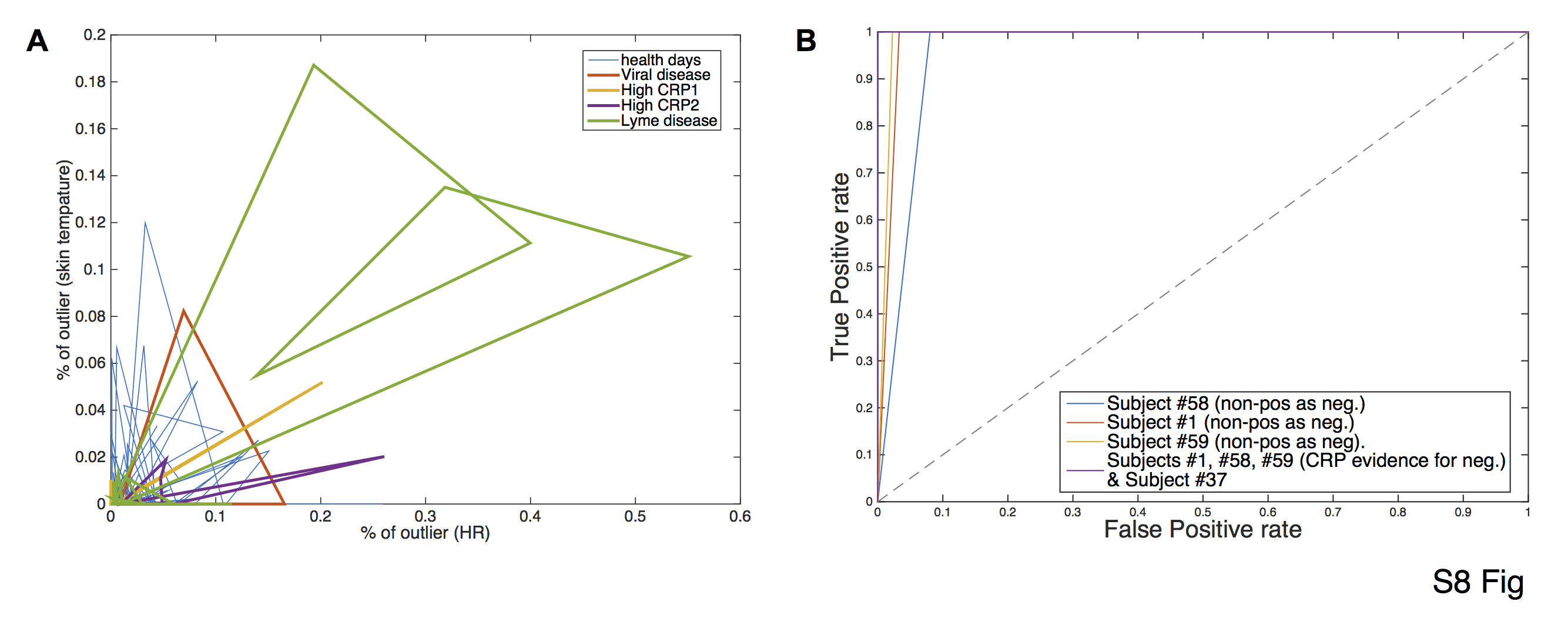

Supplement: S8 Fig — (A) Plot of fraction of outlying skin temperatures and heart rates for all 679 days of Participant #1. Connection was made by time. Line color indicates the health status (blue: health days; red: viral disease; yellow: high CRP event 1; purple: high CRP event 2; green: Lyme disease). (B) ROC curves showing classification power of the COH method in distinguishing the sick periods from the health periods. For each individual, two ROC curves are shown based on different definition of the negative set: (1) negative set was defined as days with normal CRP measurements (Participants #1, #58 and #59 and #37: purple solid line, AUC = 1); (2) negative set includes all the days in the measuring period which are not include in the positive set (Participant #1: red solid line, AUC = 0.983; Participant #58: blue solid line, AUC = 0.960; Participant #59: yellow solid line, AUC = 0.989; Participant #37: purple solid line, AUC = 1). (TIFF) [file pbio.2001402.s008.tiff]
